# Identification and functional characterization of T-cell exhaustion-associated lncRNA AL031775.1 in osteosarcoma: a novel therapeutic target

**DOI:** 10.3389/fimmu.2025.1517971

**Published:** 2025-02-24

**Authors:** Yameng Wang, Jinghong Yuan, Keying Guo, Zhuoer Zhang, Junchao Zhu, Shahrzad Arya, Guowen Huang, Shengqin Li, Qi Chen, Xijuan Liu, Jingyu Jia

**Affiliations:** ^1^ Department of Orthopedics, The Second Affiliated Hospital of Nanchang University, Nanchang, Jiangxi, China; ^2^ The Second Affiliated Hospital of Nanchang University, Institute of Orthopaedics of Jiangxi Province, Nanchang, Jiangxi, China; ^3^ The Second Affiliated Hospital of Nanchang University, Jiangxi Provincial Key Laboratory of Spine and Spinal Cord Disease, Nanchang, Jiangxi, China; ^4^ Department of Orthopedics, The Second Affiliated Hospital, Jiangxi Medical College, Nanchang University, Nanchang, Jiangxi, China; ^5^ Department of Bone and Soft Tissue Tumors, Jiangxi University of Traditional Chinese Medicine, Nanchang, Jiangxi, China; ^6^ Department of Surgery, Cedars-Sinai Medical Center, Los Angeles, CA, United States; ^7^ Department of Obstetrics and Gynecology, First Affiliated Hospital of Nanchang University, Nanchang, Jiangxi, China; ^8^ Department of Pediatrics, The Second Affiliated Hospital of Nanchang University, Nanchang, Jiangxi, China

**Keywords:** osteosarcoma, T-cell exhaustion, lncRNA, tumor, immune

## Abstract

**Background:**

Osteosarcoma, an aggressive bone malignancy predominantly affecting children and adolescents, presents significant therapeutic challenges with a 5-year survival rate below 30% in metastatic cases. T-cell exhaustion, characterized by the overexpression of immune checkpoint molecules, contributes to osteosarcoma progression and immune evasion. Although targeting these inhibitory pathways has shown potential in restoring T-cell activity, the molecular regulators of T-cell depletion in osteosarcoma are poorly understood.

**Methods:**

This study employed comprehensive bioinformatics analyses on osteosarcoma samples from the TARGET database, combined with normal tissue data from the GTEx database, to identify T-cell exhaustion-associated genes and their co-expressed long non-coding RNAs (lncRNAs). Gene ontology and KEGG pathway analyses were used to elucidate immune-related pathway enrichments. A six-lncRNA prognostic model was established using LASSO regression and validated in separate cohorts. Functional assays evaluated the impact of the lncRNA AL031775.1 on osteosarcoma cell behavior and T-cell function.

**Results:**

Twenty-four key T-cell exhaustion-related genes were identified and significantly enriched in immune-related pathways, indicating their importance in the osteosarcoma immune microenvironment. The constructed six-lncRNA model stratified patients by survival prognosis, showing robust predictive performance across cohorts. Among the six identified lncRNAs, AL031775.1 is notably downregulated in osteosarcoma patients and significantly promotes osteosarcoma cell proliferation, migration, and invasion while contributing to T-cell exhaustion. In T cells, downregulation of AL031775.1 impairs antitumor immunity, upregulates immune checkpoint molecules LAG3, PD1, and CTLA4, and diminishes T-cell cytotoxic activity against tumor cells.

**Conclusion:**

This study identifies a novel six-lncRNA prognostic model and highlights the therapeutic potential of AL031775.1 in managing osteosarcoma by enhancing T-cell immunity and counteracting tumor progression. Targeting AL031775.1 represents a promising approach to improve immunotherapy efficacy in osteosarcoma. These findings provide critical insights into the molecular regulation of T-cell exhaustion and suggest a new avenue for therapeutic intervention.

## 1 Introduction

Osteosarcoma, a highly aggressive bone malignancy predominantly affecting children and adolescents, presents significant therapeutic challenges due to its rapid progression, high metastatic potential, and poor prognosis (1–7). Despite advancements in multimodal treatments, including surgery, chemotherapy, and radiotherapy, the 5-year survival rate for patients with metastatic osteosarcoma remains alarmingly low, at less than 30% (1, 2, 8, 9). Recent studies have revealed that the tumor immune microenvironment, particularly T-cell exhaustion, plays a pivotal role in osteosarcoma’s aggressiveness and resistance to therapy (10–14)

T-cell exhaustion occurs when T-cells become dysfunctional due to prolonged exposure to antigens, resulting in a diminished ability to combat tumors (15–17). This condition is often characterized by increased levels of immune checkpoint molecules, such as PD-1, CTLA-4, and TIM-3, which hinder T-cell function and facilitate tumor immune evasion (15, 18, 19). Blocking these inhibitory receptors has shown the potential to restore T cell activity, thereby slowing cancer progression (20–22). Specifically in osteosarcoma, inhibition of TIM-3 and increased CD8+ T cell infiltration has been shown to inhibit tumor growth, suggesting the potential for immunotherapy targeting T cell exhaustion (13, 23). However, the specific pathways involved in T-cell exhaustion in osteosarcoma progression have yet to be thoroughly investigated.

Given the vital role of T-cell exhaustion in osteosarcoma, it has become particularly critical to identify its molecular regulators (e.g., T-cell exhaustion-associated genes and their co-expression factors) (7, 24, 25). Identification of these factors may provide new prognostic markers and therapeutic targets. Emerging studies have shown that long-chain non-coding RNAs (lncRNAs) are essential in regulating immune responses, cell growth, and tumor invasion (26–33). Although some lncRNAs are associated with cancer progression, their specific roles in osteosarcoma and T-cell exhaustion are not fully understood.

This study aimed to identify lncRNAs associated with T-cell exhaustion in osteosarcoma and to investigate their effects on tumor progression and immune responses. Through comprehensive bioinformatics analysis, we identified key T-cell exhaustion-related genes and constructed a six-lncRNA model for risk prediction, effectively stratifying patients by survival prognosis. Among these lncRNAs, AL031775.1 was critical in osteosarcoma progression and T-cell exhaustion. Functional experiments demonstrated that overexpression of AL031775.1 acts as a tumor suppressor, inhibiting osteosarcoma cell proliferation, migration, and invasion while preserving T-cell antitumor immunity. Our findings provide novel insights into the molecular mechanisms of T-cell exhaustion in osteosarcoma and highlight AL031775.1 as a potential therapeutic target to improve patient outcomes by enhancing antitumor immunity and countering tumor progression.

## 2 Materials and methods

### 2.1 Data collection

RNA expression profiles from normal tissues were sourced from the Genotype-Tissue Expression (GTEx) database. The fragments per kilobase of transcript per million mapped reads (FPKM) values in the expression dataset were transformed using the log2(x+0.001) method to facilitate analysis. Furthermore, RNA sequencing data, along with clinical characteristics of osteosarcoma patients, were obtained from the TARGET database. Afterward, the TARGET and GTEx datasets were combined, and batch effects were addressed using the sva package. The FPKM values were utilized as indicators of gene expression levels. In addition this study was approved by the Ethics Committee of the Second Affiliated Hospital of Nanchang University. Samples from 32 patients diagnosed with OS were collected in the Second Affiliated Hospital of Nanchang University.

### 2.2 Differentially expressed osteosarcoma-related TEXRGs

A comparative analysis was conducted between the 40 T-cell exhaustion-related genes (TEXRGs) and the genes identified within the osteosarcoma transcriptome dataset to isolate TEXRGs associated with osteosarcoma. 40 T-cell exhaustion-associated genes were obtained from the article by Zhang Z et al. (34). The differential expression analysis was carried out using the *limma* package in R, applied to the TARGET-OS and GTEx datasets, to identify differentially expressed genes (DEGs). The criteria for selection included an FDR threshold of less than 0.05 and an absolute log_2_ fold change (|log_2_FC|) greater than 2. Subsequently, an intersection of TEXRGs and DEGs was performed to identify the differentially expressed TEXRGs relevant to osteosarcoma.

### 2.3 Functional enrichment analysis

In this study, differentially expressed TEXRGs were analyzed by Kyoto Encyclopedia of Genomes (KEGG) and GO enrichment analysis using the R software packages clusterProfiler, org.Hs.eg.db, enrichplot, GOplot, and ggplot2 with a significance threshold of p < 0.05 (34–39).

### 2.4 Identification of TEXRLs and construction of risk prognostic signature

The *limma* package in R was employed to perform a co-expression analysis of osteosarcoma-associated differentially expressed TEXRGs and lncRNAs (TEXRLs) within the osteosarcoma transcriptomic dataset. This analysis aimed to identify TEXRLs relevant to osteosarcoma, utilizing screening criteria of |Pearson correlation coefficient| > 0.4 and *p* < 0.001.

To extract TEXRLs significantly associated with osteosarcoma prognosis, the survival package in R was applied, conducting univariate COX regression analysis with a significance threshold of p < 0.05, thereby calculating the hazard ratio (HR) values. Significant variables (p < 0.05) were then analyzed by the least absolute shrinkage and selection operator (LASSO) using the ‘glmnet’ software package. Candidate genes were then identified based on the optimal penalty parameter λ determined by the 1-SE (standard error) criterion (40–43), which helped reduce the risk of overfitting while determining the optimal number of TEXRLs for inclusion in the model development. The dataset was divided into training and testing cohorts to assess the model’s accuracy. A risk prognosis model was constructed for the entire dataset, as well as separately for the training and testing groups. The expression levels of the osteosarcoma prognosis TEXRLs were multiplied by their respective regression coefficients and summed to calculate the sample risk score. Subsequently, the samples from the overall dataset, training, and testing groups were stratified into high- and low-risk categories based on the median risk score. Riskscore =(-2.300*AC090559.2)+(-2.574*AL031775.1)+(2.146*LINC01060)+(-6.282*LINC02777)+(-2.543*PSMB8-AS1)+(-1.268*AC135178.4).

### 2.4 Validation of risk prognostic signature

The analysis of risk curves, survival outcomes, receiver operating characteristic (ROC) curves, and independent prognostic evaluations was performed on the risk prognostic models across the entire sample, as well as the training and testing cohorts. The statistical software R was employed to produce both the survival status map and the risk heatmap associated with the prognostic model, facilitating the assessment of differences in patient survival times and overall survival prognoses across high- and low-risk groups. To construct survival curves, the *survival* and *survminer* packages in R were utilized, while the *survival*, *survminer*, and *timeROC* packages were applied to generate ROC curves. Furthermore, the *survival* package in R was leveraged to carry out independent prognostic evaluations through univariate and multivariate Cox regression analyses, aiming to ascertain whether the risk score could function as an independent prognostic factor (44, 45).

### 2.5 Tumor microenvironment analysis

The analysis of the tumor microenvironment concerning the osteosarcoma transcriptome data was performed using the *limma*, *CIBERSORT*, and *estimate* packages in R, which generated immune scores, stromal scores, and comprehensive scores for each patient diagnosed with osteosarcoma. Furthermore, the *limma* and *ggpubr* packages in R were applied to evaluate the variations in immune, stromal, and overall scores across the risk prognostic model within the entire cohort of samples.

### 2.6 Single-sample gene set enrichment analysis

This study utilized the GSVA, limma, and GSEABase packages in R to calculate enrichment scores pertaining to immune cell types and immune functionality based on the osteosarcoma transcriptome data. Additionally, the *limma*, *reshape2*, and *ggpubr* packages in R were employed to investigate the differences in immune cell populations and immune function across the risk prognosis model applied to the entire sample cohort.

### 2.7 ScRNA-Seq data processing and analysis

Single-cell RNA sequencing (scRNA-seq) data were taken from the GSE162454 dataset in the GEO database, containing a total of 6 osteosarcoma samples. Standardized data were pre-processed by the ‘seurat’ software package (version 4.0). Strict quality control measures were used to exclude cells with less than 300 or more than 2000 genes detected. To mitigate batch effects, the ‘CAA’ software package was used. After filtering, a total of 46,544 cells were available for subsequent analyses. Subsequently, primary cell cluster analysis was performed using the FindClusters function of the Seurat package (resolution = 0.15), and the visual clustering results were presented through performing uniform manifold approximation and projection (UMAP) dimension reduction analysis (46). For other specific parameters, please refer to the TISCH2 database (http://tisch.comp-genomics.org/).

### 2.8 Overexpression of h-AL031775.1 and siRNA of h-AL031775.1

Overexpression plasmids for h-AL031775.1 were constructed by Hans Group Holdings Co., Ltd.h-AL031775.1 siRNA (CCCTTTACATTTCCCACTT) and NC siRNA (AAGTCGGGTC

AAGAGAAGC) were purchased from Guangzhou RiboBio Co., Ltd.

### 2.9 Cell lines and culture

Human osteoblast cell line hFOB 1.19 from Procell Life Co., Ltd. was cultured at 33.5°C~34°C, and the medium used was special medium for osteoblasts from Procell Life Co (Wuhan, China). The osteosarcoma cell lines MG63, U2OS, and 143B were obtained from the National Collection of Authenticated Cell Cultures (Shanghai, China). Osteosarcoma cells were cultured in complete DMEM supplemented with 10% fetal bovine serum (FBS) and 1% penicillin/streptomycin at 37°C in a humidified atmosphere with 5% CO_2_. Osteoblast cell lines were maintained in the same medium but cultured at 34°C under identical atmospheric conditions.

### 2.10 Quantitative real-time PCR

Total cellular RNA was isolated using the Trizol method and then reverse transcribed into complementary DNA (cDNA) with the aid of a TAKARA commercial kit (Cat. No. RR047A). This cDNA served as the template for real-time quantitative polymerase chain reaction (RT-qPCR), utilizing TAKARA’s qPCR mix (Cat. No. RR420A). Gene expression levels were quantified using the 2^(-ΔΔCt) comparative method for relative quantification. Primer sequences are shown in **Supplementary Table S1**. Primer sequences are synthesized by Guangzhou RiboBio Co., Ltd. (China).

### 2.11 Cell proliferation assays

The proliferative potential of osteosarcoma cells was evaluated through EdU incorporation and CCK-8 assays. For the EdU assay, cells were seeded into 96-well plates at a density of 2×10^4^ cells per well. After 8-hour incubation at 37°C, cells were labeled, fixed, and stained in accordance with the YF^®^594 Click-iT EDU staining kit protocol (UE, Shanghai, China), then imaged using a fluorescence microscope.

In the CCK-8 assay, 5000 cells per well were seeded in a 96-well plate, and at designated intervals (0 h, 24 h, 48 h, and 72 h), 10% CCK-8 solution (TransGen, Beijing, China) was added. Following a 2-hour incubation at 37°C, absorbance at 450 nm was measured via spectrophotometry to assess cell proliferation rates.

### 2.12 Wound healing assays

Osteosarcoma cells transfected with plasmids or siRNAs, along with their respective controls, were seeded into six-well plates. Upon reaching 90% confluency, a sterile pipette tip was used to create a scratch in the cell monolayer. Cells were then cultured in serum-free medium for 12 hours. The scratch area was photographed at 0 hours and after 12 hours using an inverted microscope to analyze cellular migration.

### 2.13 Transwell migration assays

To evaluate migratory potential, Transwell assays were performed using 24-well chambers. Osteosarcoma cells were seeded in the upper chamber at a density of 2×10^4^ cells in 200 µL of serum-free medium, while the lower chamber was filled with 600 µL of complete medium supplemented with 20% FBS as a chemoattractant. After an incubation period of 48–72 hours, cells that had migrated through the membrane into the lower chamber were fixed in 4% paraformaldehyde and stained with crystal violet for quantification.

### 2.14 Cell apoptosis assays

Apoptosis was assessed using a suspension of 10^4 cells per milliliter, seeded in a 6-well plate. Upon reaching 60-70% confluence, cells were harvested, washed with chilled phosphate-buffered saline (PBS) without Ca²^+^ and Mg²^+^, and centrifuged. Cells were then stained with Annexin V and Propidium Iodide (PI) in the dark for 15 minutes. Following the addition of 400 µL of binding buffer, apoptotic status was analyzed by flow cytometry to quantify apoptotic populations.

### 2.15 Animal models

The Ethics Committee of Nanchang University approved all experimental procedures involving animals, and all procedures followed the ARRIVE guidelines. Male nude mice, 4 weeks of age, were obtained from Jicui Pharmachem Biotechnology Co Ltd (Jiangsu, China). The skin of nude mice was disinfected in a sterile environment, and the insulin injection needle was used to inject two to three million cells into the axilla of nude mice, and the injection site was disinfected by slowly withdrawing the needle. The health status and tumor growth of the nude mice were monitored daily after the operation.

### 2.16 T cell killing assay

Human peripheral blood mononuclear cells were extracted using the Human Peripheral Blood Mononuclear Cell Extraction Kit (Solarbio, Beijing). After extraction, erythrocytes were lysed on ice using Lysate Red (Tiangen, Beijing), and then activated for 48h in a 24-well plate incubated with CD3/CD28 cytokines (Thermofisher, USA) on the previous day, and then activated using 1640 complete medium containing IL-2 cytokines (Thermofisher, USA) medium containing IL-2 cytokines (Gibco, USA) was used for incubation. The optimal killing ratios of 1:1, 1:2, 1:4, and 1:8 were used for the detection of tumor cells and T cells, respectively, and the killing ability of T cells was determined by using CCK-8 to detect the OD value of tumor cells after co-cultivation in 96-well plates.

### 2.17 Immune checkpoint assay

Add 5×10^6^ T cells into the flow tube, add 0.5% BSA to wash the cells, add CD3, CD4, CD8, LAG3, CTLA-4, PD1 antibody (Biolegend, Beijing), avoid light for 30min at room temperature, 0.5% BSA to wash the cells once, centrifuge and add 200ul PBS to use flow cytometry for detection.

### 2.18 Statistical analysis

Statistical analyses were performed using R software (version 4.2.3). To analyze the data expressing differences, we used the t-test and ANOVA. ROC curves and area under the ROC curve (AUC) were calculated using MedCalc for Windows version 19.3.0 (MedCalc Software, Ostend, Belgium). Data are presented as mean ± SD *n*=3. ns was considered not statistically significant, and less than 0.001 (***), 0.01 (**), and 0.05 (*) were considered statistically significant.

All analyses were performed using Flowjo, GraphPad Software, and Xiantao Academic.

## 3 Result

### 3.1 Osteosarcoma-related differentially TEXRGs and functional enrichment analysis

**Figure 1:**
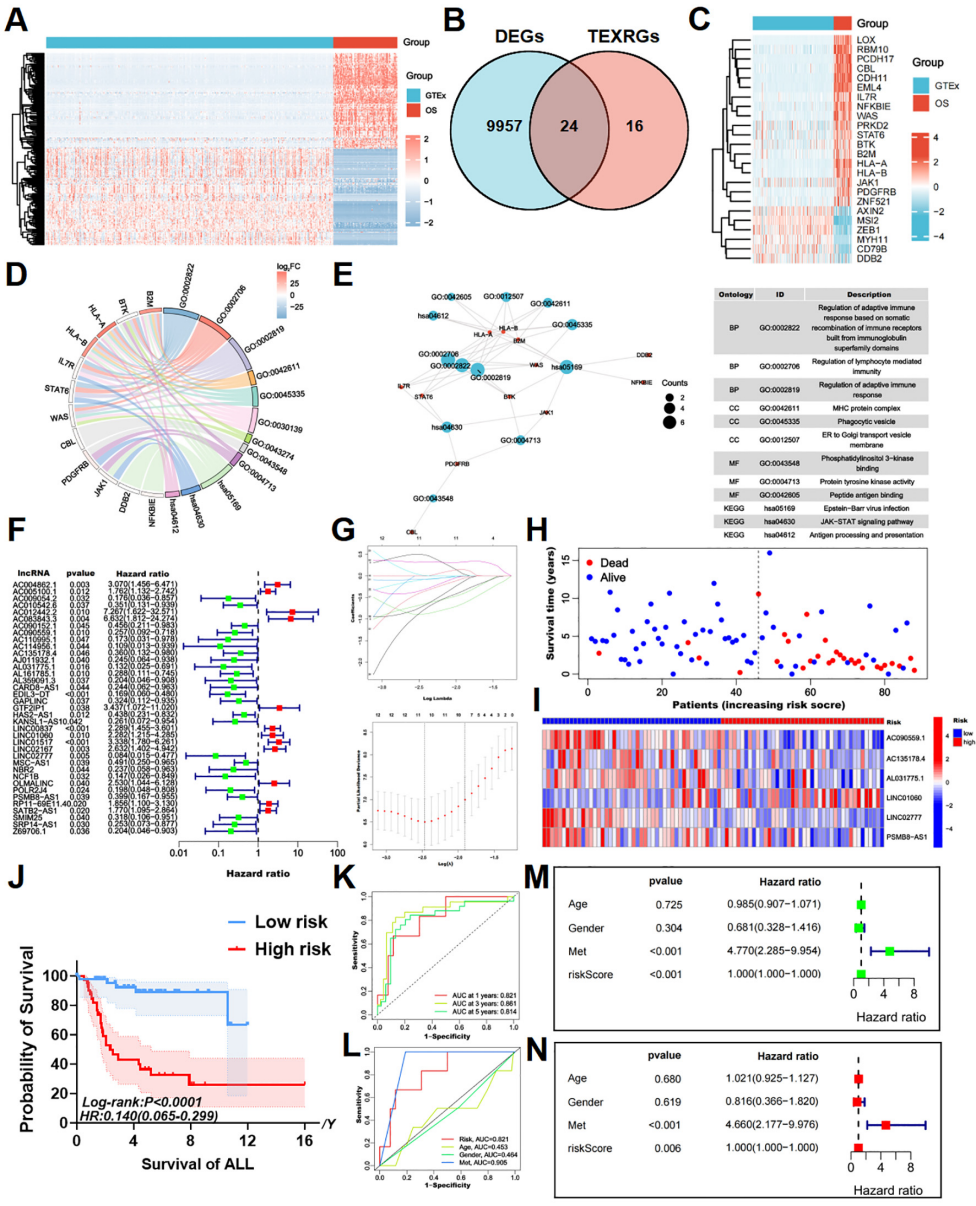
Identifying T cell exhaustion-associated genes in osteosarcoma and constructing a risk prognostic model. **(A)** The heatmap of DEGs between TARGET-OS and GTEx databases, with elevated expression depicted in red and diminished expression in blue. **(B)** The intersection of DEGs and TEXRGs yielded osteosarcoma-associated differentially expressed TEXRGs. **(C)** The heatmap of osteosarcoma-associated differentially expressed TEXRGs, with heightened expression shown in red and reduced expression in blue. **(D)** The chord diagram presents the functional enrichment analysis of osteosarcoma-associated differentially expressed TEXRGs. **(E)** The functional enrichment network and table of osteosarcoma-associated differentially expressed TEXRGs. **(F)** Univariate Cox regression analysis identified 37 potential prognostic TEXRLs for osteosarcoma, comprising 12 high-risk TEXRLs and 25 low-risk TEXRLs. **(G)** LASSO regression analysis and determining the optimal penalty parameter for LASSO regression. **(H)** The survival status map and risk heatmap of risk model TEXRLs in the total sample group. **(I)** The Kaplan-Meier survival curve effectively demonstrates that patients in the red high-risk group exhibited a substantially lower overall survival rate compared to those in the blue low-risk group. **(J)** The survival analysis of the complete sample cohort (*p* < 0.001), as well as the training cohort (*p* < 0.001) and test cohort (*p* = 0.005), demonstrated significant disparities in survival outcomes between patients categorized as red high-risk and blue low-risk.**(K)** Time-dependent ROC curves, 1 year (AUC = 0.821), 3 years (AUC = 0.861), and 5 years (AUC = 0.814). **(L)** Clinical ROC curves, Risk score (AUC = 0.821), Age (AUC = 0.453), Gender (AUC = 0.464), and Met (AUC = 0.905). **(M, N)** Univariate and multivariate COX regression analyses in the total sample group.

Analysis of the TARGET dataset, which included 88 osteosarcoma patients, and the GTEx dataset, comprising 90 control samples, identified 9,981 differentially expressed genes (DEGs), including 4,979 upregulated and 5,002 downregulated genes. These findings were visualized using heatmaps generated with R programming (**Figure 1A**).

To further investigate T-cell exhaustion in osteosarcoma, we referred to a previously published study (34), which defined a T-cell exhaustion-related gene set consisting of 40 genes. By intersecting this gene set with the osteosarcoma-specific DEGs, we identified 24 T-cell exhaustion-related genes (TEXRGs) associated with osteosarcoma, including 18 upregulated and 6 downregulated genes (**Figures 1B, C**).

Notably, 11 of these TEXRGs were significantly enriched in pathways associated with the regulation of adaptive immune responses, particularly those involving the somatic recombination of immune receptors from immunoglobulin superfamily domains and lymphocyte-mediated immunity. KEGG and GO analysis further revealed significant enrichment in the T cell receptor binding, JAK-STAT signaling pathway and PD-L1 expression and PD-1 checkpoint pathway in cancer, highlighting the potential role of these 11 TEXRGs in tumor regulation (**Figures 1D, E**; **Supplementary Figure 5C**).

### 3.2 Construction of risk prognostic signature

Co-expression analysis of these 24 TEXRGs identified 549 lncRNAs associated with T-cell exhaustion. Through Cox regression analysis, we determined that 37 of these lncRNAs were significantly correlated with patient prognosis. These included 12 high-risk T-cell exhaustion-related lncRNAs (AC012442.2, AC083843.3, GTF2IP1, LINC01517, AC004862.1, LINC02167, OLMALINC, LINC00837, LINC01060, RP11-69E11.4, SATB2-AS1, and AC005100.1) and 25 low-risk T-cell exhaustion-related lncRNAs (LINC02777, AC114956.1, AL031775.1, NCF1B, EDIL3-DT, AC110995.1, AC009054.2, POLR2J4, AL359091.3, Z69706.1, NBR2, CARD8-AS1, AJ011932.1, SRP14-AS1, AC090559.1, KANSL1-AS1, AL161785.1, SMIM25, GAPLINC, AC010542.6, AC135178.4, PSMB8-AS1, HAS2-AS1, AC090152.1, and MSC-AS1) (**Figure 1F**; **Supplementary Figure 5A**).

**Figure 2:**
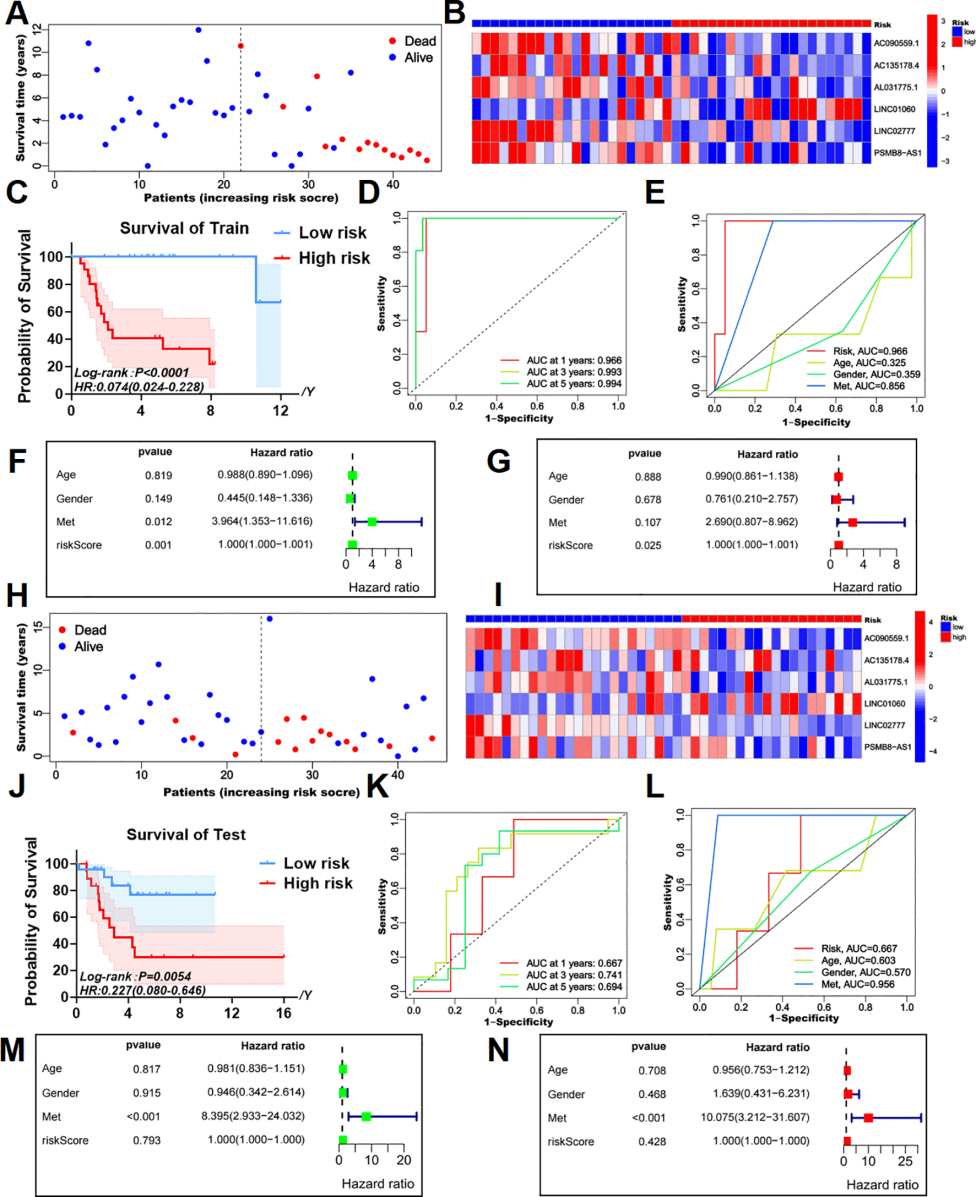
Survival prediction validation of risk models in training and testing groups. **(A, B)** The survival status map and risk heatmap of risk model TEXRLs in the training group. **(C)** In the training group, the Kaplan-Meier survival curve effectively demonstrates that patients in the red high-risk group exhibited a substantially lower overall survival rate compared to those in the blue low-risk group. **(D)** Time-dependent ROC curves in the training group, 1 year (AUC = 0.966), 3 years (AUC = 0.993), and 5 years (AUC = 0.994). **(E)** Clinical ROC curves in the training group, Risk score (AUC = 0.966), Age (AUC = 0.325), Gender (AUC = 0.359), and Met (AUC = 0.856). **(F, G)** Univariate and multivariate COX regression analyses in the training group. **(H, I)** The survival status map and risk heatmap of risk model TEXRLs in the test group. **(J)** In the test group, the Kaplan-Meier survival curve effectively demonstrates that patients in the red high-risk group exhibited a substantially lower overall survival rate compared to those in the blue low-risk group. **(K)** Time-dependent ROC curves in the test group, 1 year (AUC = 0.667), 3 years (AUC = 0.741), and 5 years (AUC = 0.694). **(L)** Clinical ROC curves in the test group, Risk score (AUC = 0.667), Age (AUC = 0.603), Gender (AUC = 0.570), and Met (AUC = 0.956). **(M, N)** Univariate and multivariate COX regression analyses in the test group.

Using LASSO regression analysis, we further identified six key T-cell exhaustion-related lncRNAs as the optimal feature set (**Figure 1G**). Importantly, these six lncRNAs (LINC01060, AC090559.1, AC135178.4, AL031775.1, LINC02777, and PSMB8-AS1) successfully constructed a robust risk prediction model for osteosarcoma (**Figure 2G**). Using the prognostic model formula, we calculated risk scores for each sample, stratifying the cohort into red high-risk (N = 42) and blue low-risk (N = 46) groups. The training cohort was further divided into red high-risk (N = 22) and blue low-risk (N = 22) groups, while the testing cohort consisted of red high-risk (N = 20) and blue low-risk (N = 24) groups.

### 3.3 Risk prognostic signature predicts the prognosis of patients with osteosarcoma

Survival status maps for the entire cohort, as well as the training and testing groups, indicated a marked increase in mortality rates as patients transitioned from blue low-risk to red high-risk categories (**Figures 1H**, **2A, H**). Risk heatmaps revealed that the expression levels of the high-risk TEXRL LINC01060 consistently increased from low-risk to red high-risk groups, while blue low-risk TEXRLs, including AC090559.1, AC135178.4, AL031775.1, LINC02777, and PSMB8-AS1, exhibited a progressive decline (**Figures 1I**, **2B, I**).

Survival analyses demonstrated significant differences in outcomes between high-risk and low-risk patients for the complete sample cohort [*p* < 0.0001, HR=0.140(0.065-0.299)], training cohort [*p* < 0.0001, HR=0.074(0.024-0.228)], and testing cohort [*p* =0.0054, HR=0.227(0.080-0.646)] (**Figures 1J**, **2C, J**). There was also a significant difference in prognosis between high- and low-risk patients in our own cohort sample (**Supplementary Figure 5B**). The receiver operating characteristic (ROC) curve for the entire sample cohort showed areas under the curve (AUC) of 0.821 at 1 year, 0.861 at 3 years, and 0.814 at 5 years (**Figure 1K**). The training cohort’s ROC curve yielded higher AUCs: 0.966 at 1 year, 0.993 at 3 years, and 0.994 at 5 years (**Figure 2D**). The testing cohort demonstrated lower AUCs: 0.667 at 1 year, 0.741 at 3 years, and 0.694 at 5 years (**Figure 2K**). Additionally, the ROC curves for metastasis (AUC = 0.905) and risk score (AUC = 0.821) in the complete cohort indicated strong predictive capacity (**Figure 1L**). In the training cohort, metastasis (AUC = 0.856) and risk score (AUC = 0.966) also showed high predictive values (**Figure 2E**). The test cohort exhibited AUCs of 0.956 for metastasis, 0.667 for risk score, and 0.603 for age (**Figure 2L**).

Univariate independent prognostic analysis revealed that both the risk score (*p* < 0.001, HR = 1.000) and tumor metastasis (*p* < 0.001, HR = 4.770) are independent prognostic factors (**Figure 1M**). Multivariate analysis confirmed the risk score (*p* = 0.006, HR = 1.000) and tumor metastasis (*p* < 0.001, HR = 4.660) as independent prognostic indicators (**Figure 1N**). In the training cohort, univariate analysis identified the risk score (*p* = 0.001, HR = 1.000) and tumor metastasis (*p* = 0.012, HR = 3.964) as significant prognostic determinants (**Figure 2F**). Multivariate analysis further established the risk score (*p* = 0.025, HR = 1.000) as an independent prognostic factor (**Figure 2G**). In the test cohort, univariate analysis indicated that tumor metastasis (*p* < 0.001, HR = 8.395) serves as an independent prognostic factor (**Figure 2M**). Moreover, multivariate analysis confirmed tumor metastasis (*p* < 0.001, HR = 10.075) as a significant independent prognostic indicator (**Figure 2N**). Collectively, our risk prognostic model suggests that both the risk score and tumor metastasis may serve as independent high-risk prognostic factors for osteosarcoma patients.

### 3.4 Risk prognostic signature guides the immune microenvironment of patients with osteosarcoma

**Figure 3:**
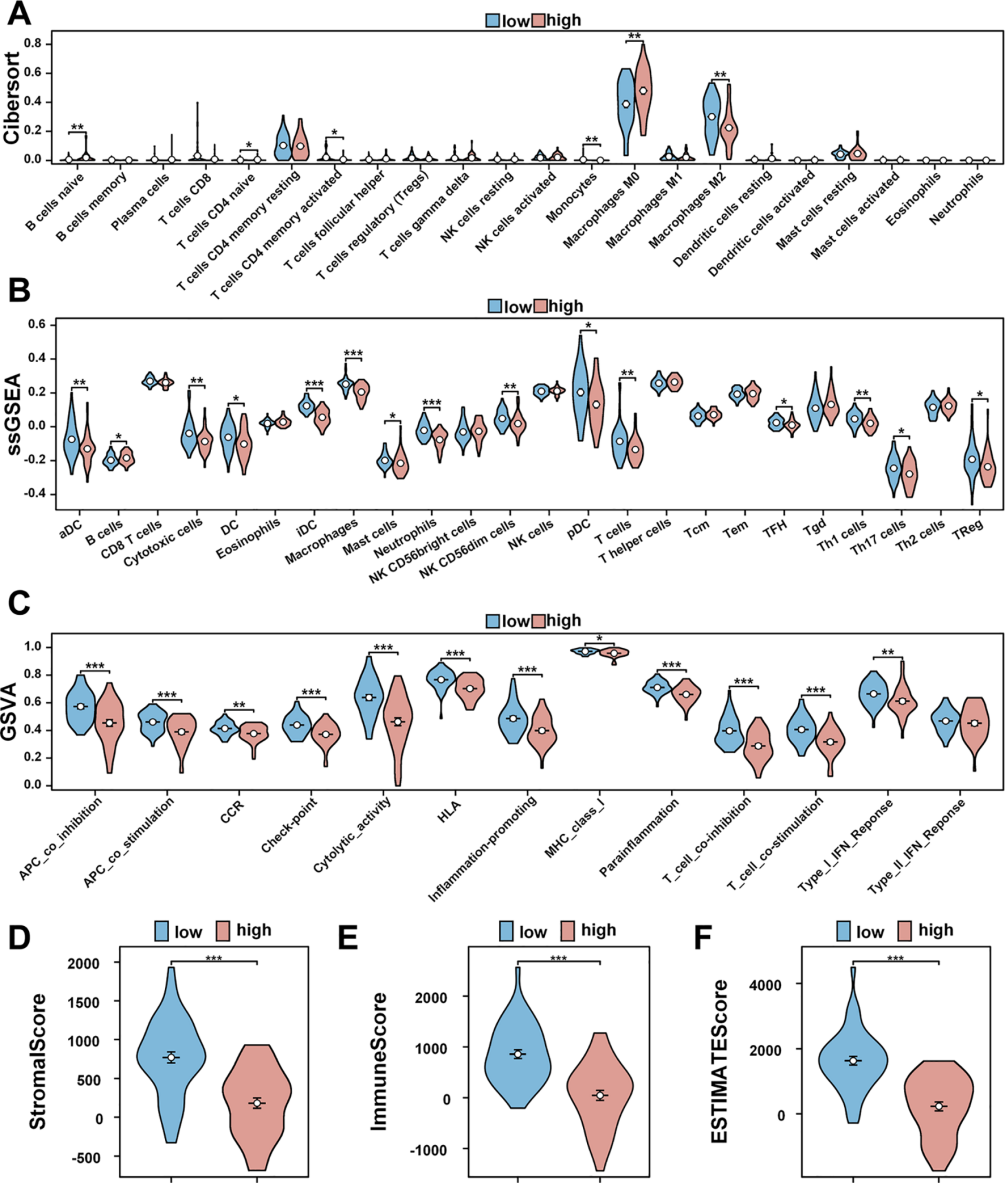
Immune cell infiltration and immune microenvironment analysis of patients with osteosarcoma. **(A)** Using the CIBERSORT package, the LM22 algorithm was used to calculate the difference in expression of 22 immune cells in the high (red) and low (blue) risk groups in the TARGET-OS database. **(B)** A violin plot of immune cell infiltration differences was analyzed using ssGSEA. **(C)** A violin plot of immune function differences was analyzed using GSVA. **(D–F)** Violin plots of the differences in StromalScore, ImmuneScore, and ESTIMATEScore between red high-risk and blue low-risk groups. *P<0.05, **P<0.01, ***P<0.001.

CIBERSORT analysis of immune cell populations revealed a significant increase in naive B cells, naive CD4 T cells, and macrophages in the high-risk cohort (**Figure 3A**). Conversely, activated memory CD4 T cells, monocytes, and M2 macrophages were notably decreased in high-risk individuals (**Figure 3A**). Additionally, ssGSEA analysis indicated a marked downregulation of various immune cell types in the high-risk group, including activated dendritic cells (aDC), cytotoxic cells, dendritic cells (DC), immature dendritic cells (iDC), macrophages, mast cells, neutrophils, NK CD56dim cells, plasmacytoid dendritic cells (pDC), T cells, T follicular helper cells (TFH), Th1 cells, and regulatory T cells (T-Reg) (**Figure 3B**). B cells were significantly upregulated in this cohort (**Figure 3B**).

Comparative analysis of immune functionality showed significant downregulation of several factors in the high-risk cohort, including antigen-presenting cell (APC) co-inhibition, APC co-stimulation, chemokine receptors (CCR), immune checkpoint molecules, cytolytic activity, human leukocyte antigen (HLA), inflammation-promoting agents, major histocompatibility complex (MHC) class I, para-inflammation, T cell co-inhibition, T cell co-stimulation, and Type I interferon (IFN) response (**Figure 3C**).

Examination of the tumor microenvironment revealed notable differences in stromal cell scores (*p* < 0.001) (**Figure 3D**), immune cell scores (*p* < 0.001) (**Figure 3E**), and ESTIMATE scores (*p* < 0.001) (**Figure 3F**) when comparing red high-risk and blue low-risk groups, with the low-risk group exhibiting higher scores across these metrics (**Figures 3D–F**).

### 3.5 Single lncRNAs predict the prognosis of patients with osteosarcoma

**Figure 4:**
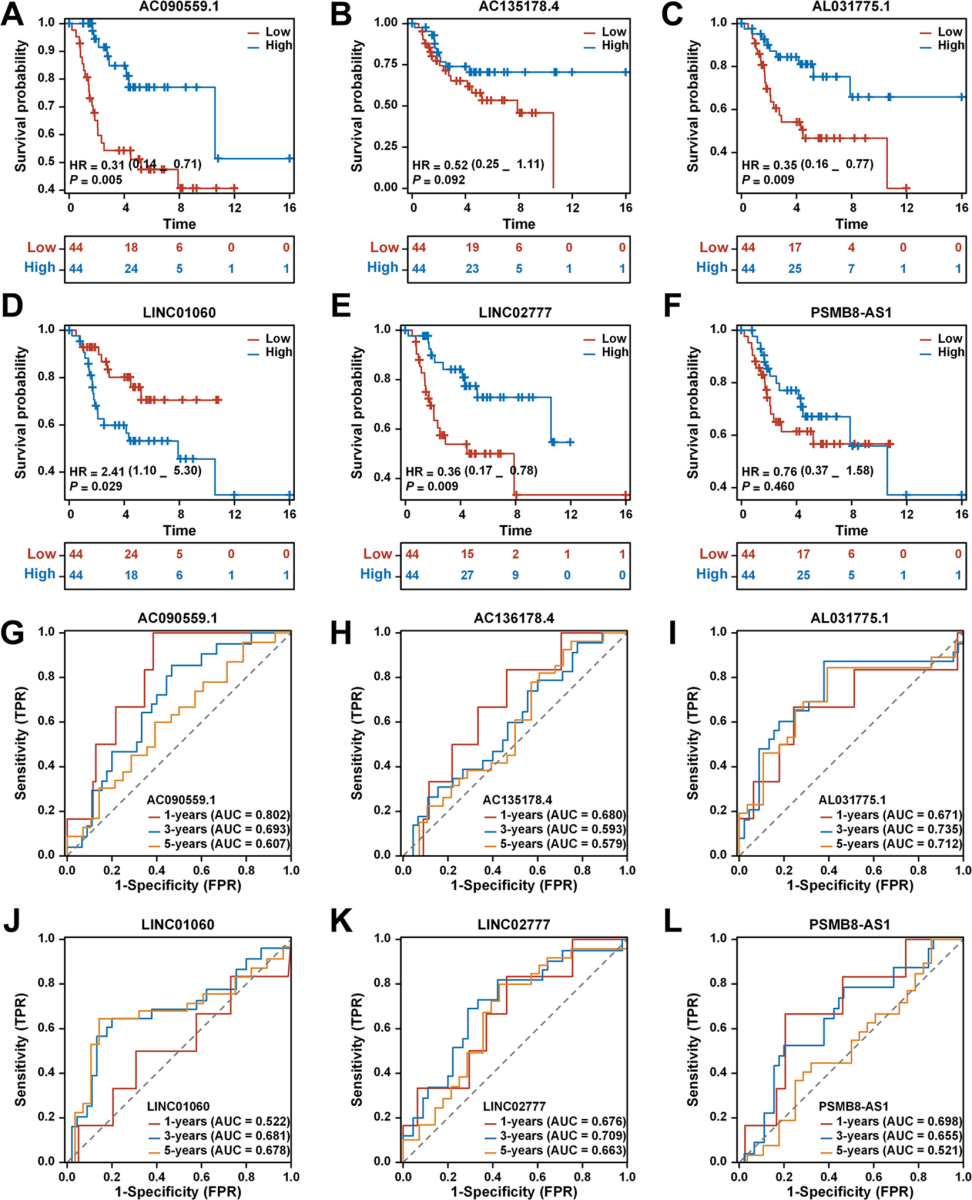
Analysis of the prognostic prediction ability of single genes from the risk model. **(A)** The effect of high AC090559.1 expression on the prognosis of osteosarcoma overall survival is statistically significant. **(B)** The effect of high AC135178.4 expression on the prognosis of osteosarcoma overall survival is statistically significant. **(C)** Kaplan-Meier survival curve analysis indicates that the expression level of AL031775.1cannot be used to predict the survival prognosis of osteosarcoma patients. **(D)** The effect of low LINC01060 expression on the prognosis of osteosarcoma overall survival is statistically significant. **(E)** The effect of high LINC02777 expression on the prognosis of osteosarcoma overall survival is statistically significant. **(F)** Kaplan-Meier survival curve analysis indicates that the expression level of PSMB8-AS1 cannot be used to predict the survival prognosis of osteosarcoma patients. **(G)** Time-dependent ROC curves of AC090559.1, 1 year (AUC = 0.802), 3 years (AUC = 0.693), and 5 years (AUC = 0.607). **(H)** Time-dependent ROC curves of AC135178.4, 1 year (AUC = 0.680), 3 years (AUC = 0.593), and 5 years (AUC = 0.579). **(I)** Time-dependent ROC curves of AL031775.1, 1 year (AUC = 0.671), 3 years (AUC = 0.735), and 5 years (AUC = 0.712). **(J)** Time-dependent ROC curves of LINC01060, 1 year (AUC = 0.522), 3 years (AUC = 0.681), and 5 years (AUC = 0.678). **(K)** Time-dependent ROC curves of LINC02777, 1 year (AUC = 0.676), 3 years (AUC = 0.709), and 5 years (AUC = 0.663). **(L)** Time-dependent ROC curves of PSMB8-AS1, 1 year (AUC = 0.698), 3 years (AUC = 0.655), and 5 years (AUC = 0.521).

Kaplan-Meier survival analysis for AC090559.1, AL031775.1, LINC01060, and LINC02777 indicated significant differences in survival outcomes between patients with blue high and red low expression levels of these lncRNAs (**Figures 4A–F**).

The ROC curve for AC090559.1 demonstrated a commendable AUC of 0.802 at 1 year, 0.693 at 3 years, and 0.607 at 5 years (**Figure 4G**). In contrast, AC136178.4 displayed lower AUC values: 0.680 at 1 year, 0.593 at 3 years, and 0.579 at 5 years (**Figure 4H**). AL031775.1 showed a better performance with AUCs of 0.671 at 1 year, 0.735 at 3 years, and 0.712 at 5 years (**Figure 4I**). LINC01060 also revealed notable AUC values of 0.522 at 1 year, 0.681 at 3 years, and 0.678 at 5 years (**Figure 4J**). LINC02777 exhibited favorable AUCs of 0.676 at 1 year, 0.709 at 3 years, and 0.663 at 5 years (**Figure 4K**). Lastly, PSMB8-AS1 indicated comparatively lower AUCs of 0.698 at 1 year, 0.655 at 3 years, and 0.521 at 5 years (**Figure 4L**).

### 3.6 Tumor tissue samples and osteosarcoma cell lines were tested for differential expression results of TEXRLs

Differential gene expression of six tumor-expressed long non-coding RNAs (TEXRLs) was assessed in 32 osteosarcoma tissue samples, with corresponding paracancerous tissues serving as controls. The analysis revealed that LINC01060, AL031775.1, LINC02777, and PSMB8-AS1 were significantly downregulated in osteosarcoma tissues compared to the control group, while AC090559.1 was significantly upregulated, and AC135178.4 showed no statistically significant difference (**Supplementary Figure 1A**). Of the four long non-coding RNAs exhibiting downregulation (AL031775.1, LINC01060, LINC02777, and PSMB8-AS1), AL031775.1 demonstrated the most significant statistical difference in RT-qPCR results compared to the control group. Additionally, survival analysis and AUC curve results further supported the superior predictive performance of AL031775.1 over LINC01060, LINC02777, and PSMB8-AS1 (**Figure 4**). Therefore, we selected AL031775.1 as the candidate gene for further investigation.

### 3.7 Downregulation of AL031775.1 markedly enhances osteosarcoma cell proliferation and migration while inhibiting apoptosis

**Figure 5:**
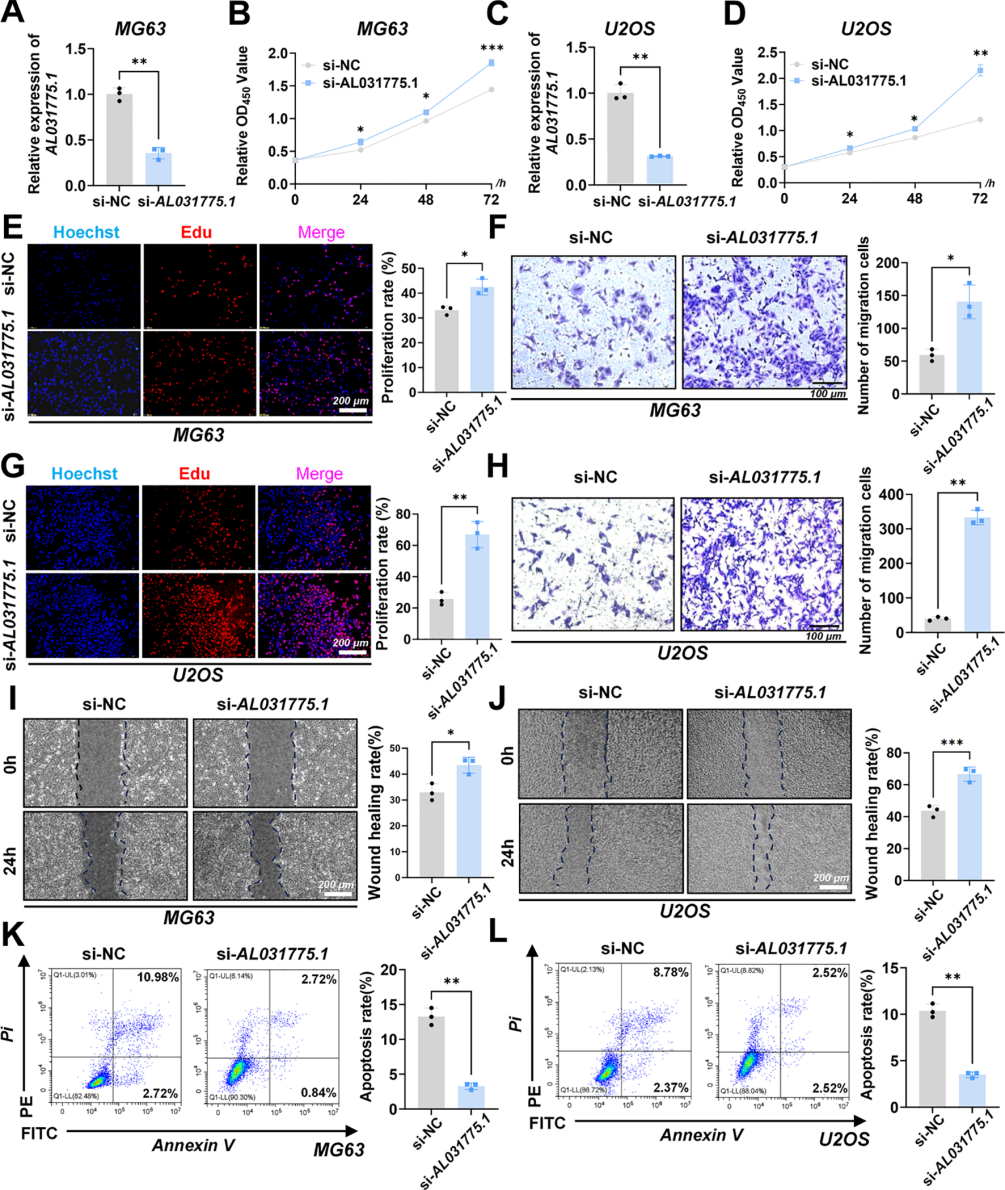
Downregulation of AL031775.1 markedly enhances osteosarcoma cell proliferation and migration while inhibiting apoptosis. **(A, C)** The expression of AL031775.1 was significantly reduced in the MG63 and U2OS cell lines. **(B, D, E, G)** CCK-8 and EdU assays demonstrated that the knockdown of AL031775.1 markedly promoted the proliferation of MG63 and U2OS cells. **(F, H)** Transwell assays indicated a significant increase in the migration capacity of MG63 and U2OS cells following AL031775.1 knockdown. **(I, J)** Scratch assays further confirmed that the migration ability of MG63 and U2OS cells was significantly enhanced after AL031775.1 knockdown. **(K, L)** Apoptosis assays revealed a substantial decrease in the apoptosis rate of MG63 and U2OS cells following the knockdown of AL031775.1. Data are presented as mean ± SD *n*=3. Statistical significance was assessed using a two-tailed Welch’s t-test. Results were considered not significant (ns) unless *P<0.05, **P<0.01, ***P<0.001, or ****P<0.0001 compared to the control group.

The expression of AL031775.1 was evaluated in three osteosarcoma cell lines, with the hFOB 1.19 cell line used as a normal control. This analysis confirmed significant downregulation of AL031775.1 in the MG63, U2OS, and 143B cell lines (**Supplementary Figure 1B**). Subsequently, the expression of AL031775.1 was inhibited using siRNA transfection (**Figures 5A, C**), and we assessed its effects on osteosarcoma cell proliferation, migration, and apoptosis. CCK-8 assay and EdU staining in the MG63 osteosarcoma cell line showed that the reduction of AL031775.1 expression significantly promoted the proliferation of osteosarcoma cells (**Figures 5B, E**), and the same results were observed in the U2OS osteosarcoma cell line (**Figures 5D, G**), Transwell assay showed that down-regulation of AL031775.1 contributed to the enhanced migratory capacity of the MG63 osteosarcoma cell line (**Figure 5F**) and the U2OS osteosarcoma cell line (**Figure 5H**), which was confirmed by a wound healing assay showing enhanced migratory activity of both the MG63 and the U2OS osteosarcoma cell lines (**Figures 5I, J**). Annexin V/PI staining analysis showed that the low expression of AL031775.1 significantly reduced apoptosis in MG63 and U2OS osteosarcoma cells (**Figures 5K, L**).

### 3.8 Upregulation of AL031775.1 significantly suppresses osteosarcoma cell proliferation and migration while promoting apoptosis

**Figure 6:**
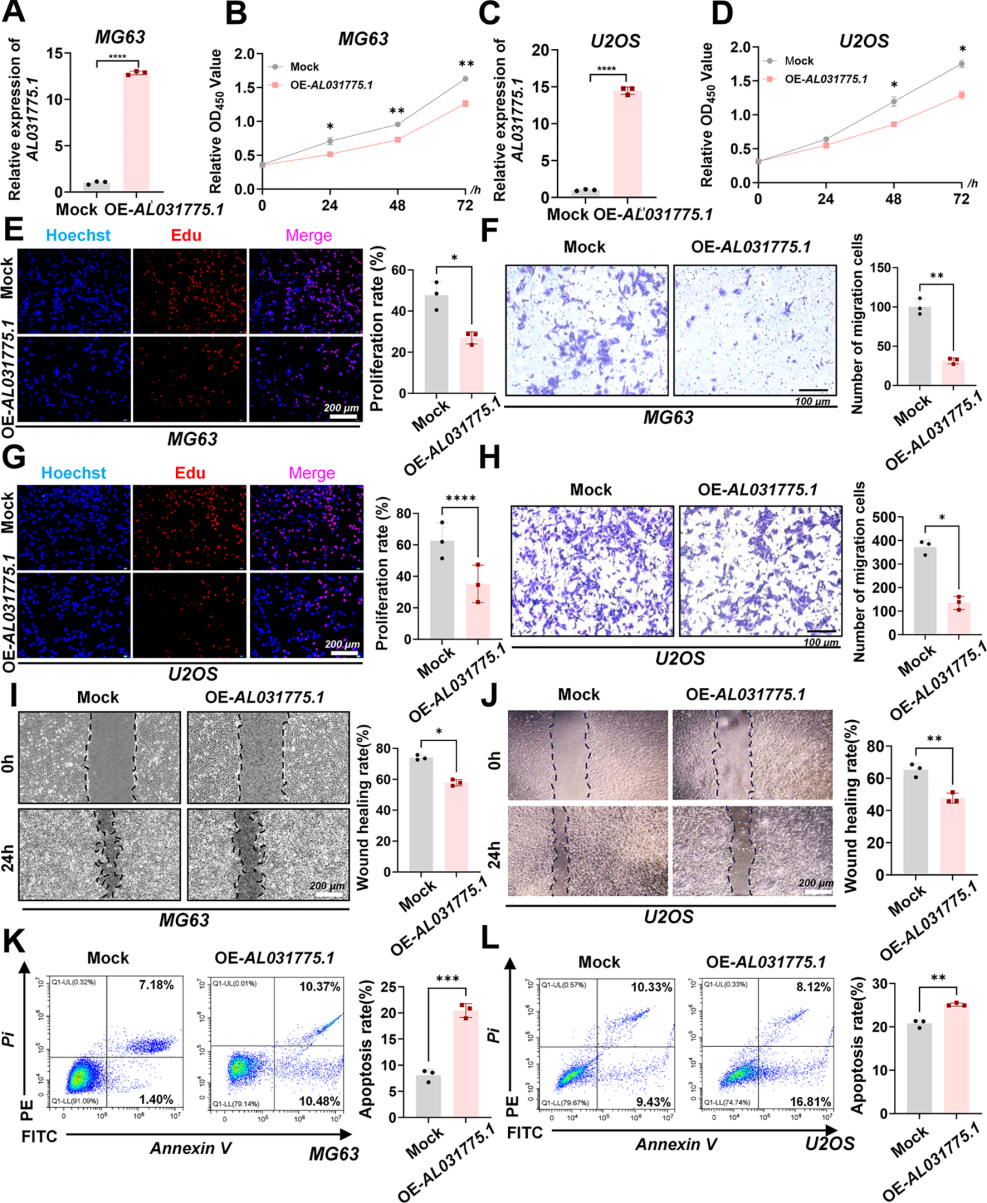
Upregulation of AL031775.1 significantly suppresses osteosarcoma cell proliferation and migration while promoting apoptosis. **(A, C)** AL031775.1 expression was significantly increased in the MG63 and U2OS cell lines. **(B, D, E, G)** CCK-8 and EdU assays demonstrated that upregulation of AL031775.1 significantly inhibited the proliferation of MG63 and U2OS cells. **(F, H)** Transwell assays indicated a significant decrease in the migration capacity of MG63 and U2OS cells following AL031775.1 upregulation. **(I, J)** Scratch assays further confirmed that the migration ability of MG63 and U2OS cells was significantly reduced after upregulation of AL031775.1. **(K, L)** Apoptosis assays revealed a significant increase in the apoptosis rate of MG63 and U2OS cells following the upregulation of AL031775.1. Data are presented as mean ± SD *n*=3. Statistical significance was assessed using a two-tailed Welch’s t-test. Results were considered not significant (ns) unless *P<0.05, **P<0.01, ***P<0.001, or ****P<0.0001 compared to the control group.

**Figure 7:**
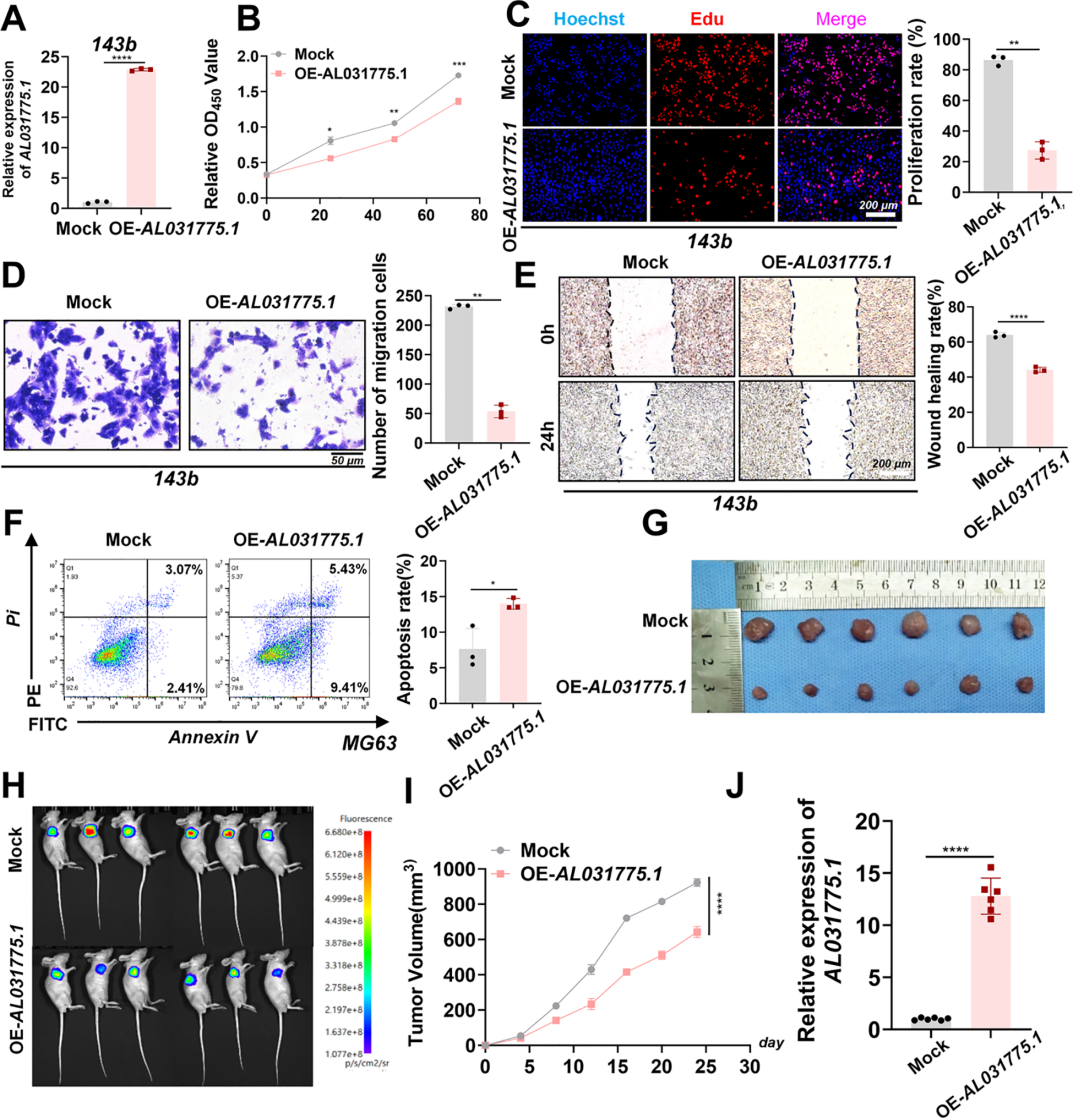
*In vitro* and *in vivo* experiments upregulated AL031775.1 for the 143b cell line. **(A)** AL031775.1 was significantly upregulated in the 143b cell line. **(B, C)** CCK-8 and Edu assay showed that upregulation of AL031775.1 significantly inhibited the proliferation of 143b cells. **(D)** Transwell assay showed that upregulation of AL031775.1 significantly decreased the migration ability of 143b cells. **(E)** The scratch assay revealed that the migratory ability of 143b cells was significantly decreased after upregulation of AL031775.1. **(F)** Apoptosis assay demonstrated that the apoptosis rate of 143b cells was significantly increased after the upregulation of AL031775.1. **(G, I)** The tumor size in nude mice was significantly reduced in the AL031775.1 overexpression group compared to the control group. **(H)** Bioluminescence imaging revealed that the fluorescence signals of tumors in the AL031775.1 overexpression group were significantly diminished compared to those in the control group. **(J)** RT-qPCR showed that AL031775.1 expression was upregulated in the nude mice of AL031775.1 overexpression. Data are presented as mean ± SD *n*=3. Statistical significance was assessed using a two-tailed Welch’s t-test. Results were considered not significant (ns) unless *P<0.05, **P<0.01, ***P<0.001, or ****P<0.0001 compared to the control group.

Overexpression vector plasmid transfection was utilized to promote AL031775.1 expression in MG63 and U2OS osteosarcoma cell lines (**Figures 6A, C**), and we subsequently assessed its effects on osteosarcoma cell proliferation, migration, and apoptosis. CCK-8 assay and EdU staining in the MG63 osteosarcoma cell line showed that increased AL031775.1 expression significantly inhibited the proliferation of osteosarcoma cells (**Figures 6B, E**), and the same results were observed in the U2OS osteosarcoma cell line (**Figures 6D, G**). Transwell assay showed that upregulation of AL031775.1 inhibited the migratory ability of the MG63 osteosarcoma cell line (**Figure 6F**) and the U2OS osteosarcoma cell line (**Figure 6H**), which was confirmed by wound healing assay showing decreased migratory activity of the MG63 and U2OS osteosarcoma cell lines((**Figures 6I, J**). Annexin V/PI staining analysis showed that the low expression of AL031775.1 significantly increased apoptosis in MG63 and U2OS osteosarcoma cells (**Figures 6K, L**) Similarly, a decrease in cell proliferation and migration was observed after overexpression of AL031775.1 in the 143b osteosarcoma cell line, along with an increase in apoptosis (**Figures 7A–F**).

### 3.9 Upregulation of AL031775.1 inhibits tumor growth in a nude mice-loaded tumor assay

To investigate the role of AL031775.1 in osteosarcoma progression, we employed a subcutaneous transplantation model in nude mice using the 143B osteosarcoma cell line. A virus-encapsulated AL031775.1 overexpression plasmid and an empty vector control plasmid were used to establish stable cell transplants. Following transduction, twelve nude mice were divided into two groups for subcutaneous tumor formation. Tumor growth differences between the groups became apparent after one week, and real-time growth curves were recorded (**Figure 7I**). Tumors in the AL031775.1 overexpression group exhibited significant growth inhibition, with reduced fluorescence signals observed *in vivo* after 24 days, indicating diminished tumor activity (**Figure 7G, H**). Additionally, RT-qPCR analysis of tumor tissues confirmed successful overexpression of AL031775.1 (**Figure 7J**).

### 3.10 ScRNA-Seq analysis in osteosarcoma

In order to gain a comprehensive understanding of the distribution of model genes in the osteosarcoma tumour microenvironment (TME), we performed an in-depth analysis of scRNA-seq data obtained from osteosarcoma patients. After stringent quality control measures, we identified a total of 46,544 cells in the osteosarcoma samples, laying the foundation for subsequent analyses. By evaluating the expression of characteristic genes, we delineated 8 major clusters in the osteosarcoma TME (**Supplementary Figures S4A, B**). The resulting t-SNE plots showed the annotations of these 8 different cell clusters, which included various cell types such as CD4Tconv, CD8Tex, Endothelial, Fibroblasts, Malignant, Mono/Macro, Osteoblasts, Plasmocytes, as **Supplementary Figure S3B** shown. In addition, the authors analyzed the percentage of different cell types among all cells and the proportion of cell type distribution in different patient samples (**Supplementary Figures S4C, D**). Correspondingly, UMAP plots and violin plots (**Supplementary Figures S4E, F**) highlight that our risk gene AL031775.1 is highly expressed in a wide range of immune cells from CD4Tconv, CD8Tex, Mono/Macro and Plasmocytes.

### 3.11 AL031775.1 regulates T cell proliferation, apoptosis, immune checkpoint expression, and cytotoxicity against osteosarcoma cells

**Figure 8:**
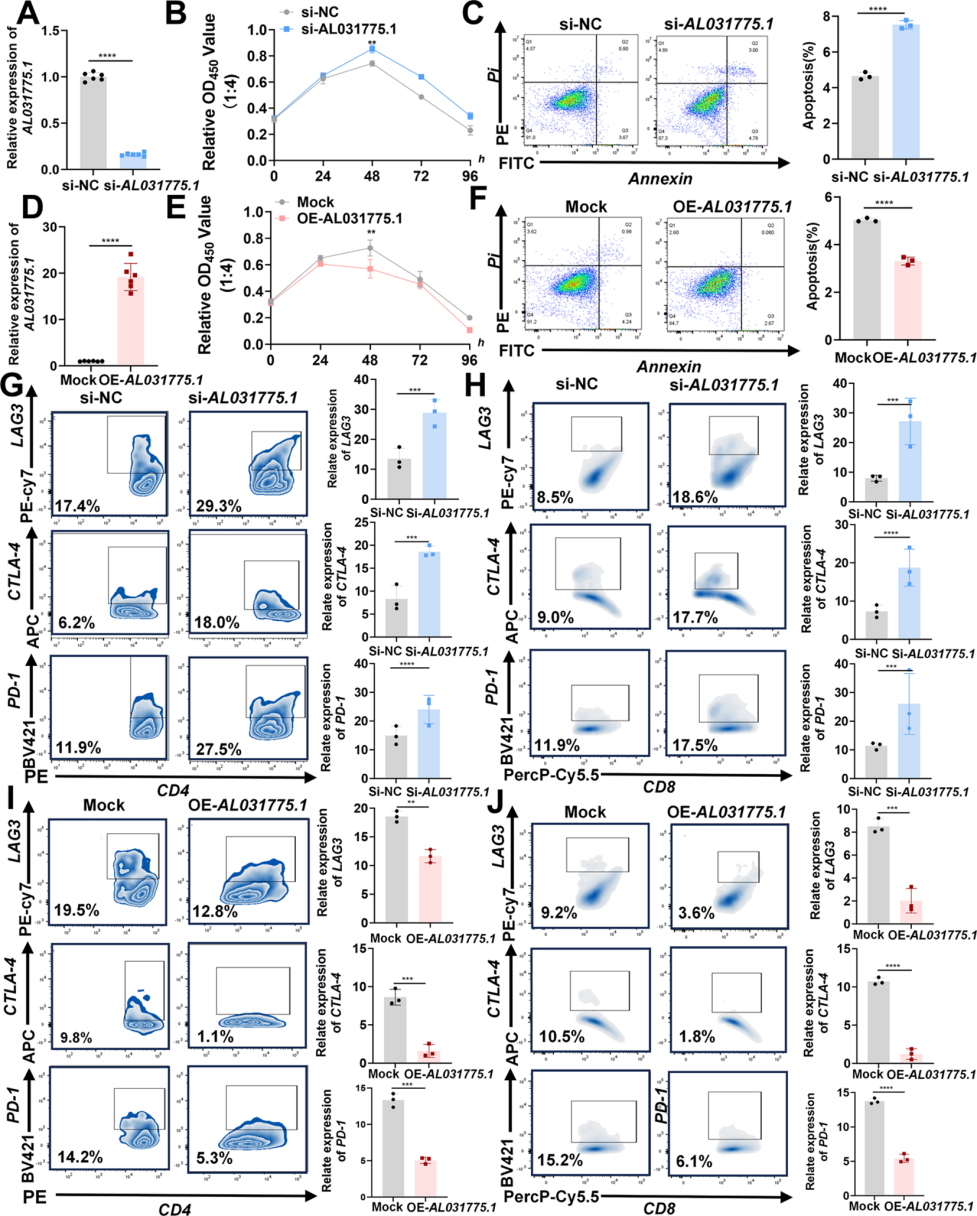
AL031775.1 regulates T-cell proliferation, apoptosis, immune checkpoint expression, and cytotoxicity against osteosarcoma cells. **(A)** AL031775.1 expression was downregulated in T cells. **(B)** The cytotoxic capacity of T cells against osteosarcoma cells was diminished following the downregulation of AL031775.1. **(C)** Apoptosis was significantly elevated in T cells after the downregulation of AL031775.1. **(G, H)** The immune checkpoints LAG3, CTLA-4, and PD-1 were upregulated in CD4 and CD8 T cells following AL031775.1 downregulation. **(D)** AL031775.1 expression was upregulated in T cells. **(E)** The cytotoxic capacity of T cells against osteosarcoma cells was enhanced following the overexpression of AL031775.1. **(F)** Apoptosis was significantly inhibited in T cells after the overexpression of AL031775.1. **(I, J)** The immune checkpoints LAG3, CTLA-4, and PD-1 were downregulated in CD4 and CD8 T cells following AL031775.1 overexpression. Data are presented as mean ± SD *n*=3. Statistical significance was assessed using a two-tailed Welch’s t-test. Results were considered not significant (ns) unless **P<0.01, ***P<0.001, or ****P<0.0001 compared to the control group.

After isolating human peripheral blood T lymphocytes, T cells were extracted and co-cultured with 143B osteosarcoma cells at varying ratios (143B cell ratios of 1:1, 1:2, 1:4, and 1:8). Following 48 hours of co-culture, the optical density (OD) values of the residual tumor cells were measured using the CCK-8 assay to assess T cell cytotoxicity. The optimal killing efficiency was observed at a 1:4 ratio (**Supplementary Figure 2**). Downregulation of AL031775.1 in T cells resulted in a significant decrease in T cell proliferation, an increase in apoptosis, and a reduced capacity for tumor cell lysis, accompanied by elevated expression of immune checkpoints LAG3, CTLA4, and PD1 (**Figures 8A–C, G, H**). Conversely, upregulation of AL031775.1 in T cells leads to opposite effects (**Figures 8D–F, I, J**).

## 4 Discussion

This study identified 24 key T-cell exhaustion-related genes in osteosarcoma through a comprehensive bioinformatics analysis, integrating data from normal muscle tissues (GTEx database) and osteosarcoma samples (TARGET database). GO and KEGG enrichment analyses demonstrated that these genes were significantly enriched in immune-related pathways, indicating their pivotal roles in modulating the tumor immune microenvironment. Notably, these genes were closely linked to T-cell exhaustion, a state that impairs effective antitumor immune responses.

To develop a prognostic model for osteosarcoma, we identified lncRNAs co-expressed with these key T-cell exhaustion-related genes and significantly associated with patient survival (47). Utilizing LASSO regression analysis, we established a six-lncRNA risk prediction model that effectively stratified patients into high- and low-risk groups with distinct survival outcomes. The model demonstrated consistent performance across both the training and validation cohorts, confirming its stability and potential for clinical application. This offers a promising tool for achieving more accurate patient stratification and providing prognostic insights in the future, fostering a sense of optimism among the audience.

Among these lncRNAs, AL031775.1 was particularly noted for its significant association with patient prognosis and T-cell dysfunction. AL031775.1 has garnered increasing attention in oncology research, with several studies highlighting its association with tumor progression (48–50). Chen et al. (49) developed a ferroptosis-related lncRNA signature for predicting prognosis in bladder cancer patients, which included AL031775.1 as a key component. Similarly, Tong et al. (48) constructed an EMT-related lncRNA signature for bladder cancer, also incorporating AL031775.1, underscoring its relevance in tumor-related pathways. Furthermore, Ni et al. (50) identified AL031775.1 as part of a cuproptosis-related lncRNA signature used to predict prognosis and immune landscape in osteosarcoma patients. Although these studies consistently associate AL031775.1 with tumorigenesis, none have experimentally validated its direct effects on osteosarcoma cell proliferation, invasion, migration, or apoptosis. Additionally, AL031775.1’s potential role in T-cell exhaustion has not yet been explored. This gap suggests an opportunity to investigate the functional and mechanistic implications of AL031775.1 in osteosarcoma and its impact on the tumor immune environment. Our studies indicated that overexpression of AL031775.1 inhibited osteosarcoma cell growth, migration, and survival. In a nude mice model, elevated expression of AL031775.1 significantly reduced tumor growth, indicating its tumor-suppressive effect.

In the immune infiltration analysis, we found that the immune cell infiltration of osteosarcoma patients in the high-risk group was significantly lower than in the low-risk group. In particular, the high-risk group exhibited lower levels of T cells, TH1 cells, and dendritic cells (DCs). This result suggests that the high-risk group had a more immunosuppressive tumor environment with reduced immune cell infiltration. Reduced T cells and TH1 cells weaken adaptive immune responses, whereas reduced DCs may further weaken immune recognition. This suggests that high-risk patients may have a poorer prognosis due to diminished immune surveillance and reduced immune defense. These findings underscore the potential clinical utility of the six-lncRNA model in predicting patient outcomes and guiding treatment decisions, reassuring the audience about its practical application.

To further investigate the role of AL031775.1 in T-cell function, we performed low- and overexpression experiments in T-cells with AL031775.1. Knockdown of AL031775.1 resulted in decreased T-cell proliferation, increased apoptosis, and elevated immune checkpoints LAG3, PD1, and CTLA4 levels. In contrast, T-cell exhaustion markers were reduced when AL031775.1 was overexpressed. In addition, T-cells with reduced AL031775.1 expression showed decreased cytotoxicity against osteosarcoma cells, suggesting a role for AL031775.1 in preventing T-cell exhaustion and maintaining T-cell function.

The observed increase in immune checkpoint expression highlights the clinical relevance of AL031775.1, as checkpoint inhibition has emerged as a key therapeutic strategy to reverse T-cell exhaustion and restore antitumor immunity. Thus, targeting AL031775.1 may represent a promising approach to modulate T-cell function and enhance the efficacy of immunotherapeutic interventions in cancer treatment. Collectively, our study provides novel insights into the mechanistic role of lncRNAs in regulating T-cell function and presents AL031775.1 as a potential therapeutic target for enhancing antitumor immunity in osteosarcoma.

In conclusion, our study highlights the pivotal role of T-cell exhaustion-related genes and their associated lncRNAs in shaping the immune landscape of osteosarcoma. We developed a robust six-lncRNA risk prediction model that accurately stratifies patients and provides valuable prognostic insight. AL031775.1 plays a critical role in T-cell function and osteosarcoma progression. Low expression of AL031775.1 in tumor cells contributes to cell proliferation, reduces apoptosis, and enhances metastatic properties. In contrast, low expression in T cells promotes T-cell exhaustion and impairs their ability to target tumor cells. Low levels of AL031775.1 in tumor tissues may promote osteosarcoma growth and invasion and impair immune-driven tumor suppression. Targeting AL031775.1 holds potential as a promising therapeutic strategy for osteosarcoma.

## 5 Limitations

The present study has several limitations. Although the effect of AL031775.1 on tumor growth was preliminarily validated using a nude mouse model, the lack of a functional immune system in these mice limits the ability to assess its role in modulating T-cell-mediated immune responses *in vivo*. Specifically, the potential effects of AL031775.1 on immune checkpoint regulation and the enhancement of T-cell cytotoxicity against tumor cells remain unclear. Future investigations utilizing BALB/c mice or other immunocompetent models are essential to confirm these findings.

In addition, the molecular mechanisms underlying AL031775.1-mediated regulation of immune checkpoint molecules and its interactions with other signaling pathways remain incompletely understood. This knowledge gap restricts a deeper understanding of its functional roles and therapeutic potential in osteosarcoma.

Moreover, this study primarily focused on T cells and their interactions within the osteosarcoma microenvironment. Other key immune components, such as macrophages, B cells, and natural killer cells, were not extensively evaluated, despite GSEA analysis suggesting significant differences in their involvement between high- and low-risk groups. Future studies should explore the contributions of these immune cells to provide a more comprehensive understanding of the osteosarcoma immune landscape.

## Data Availability

The original contributions presented in the study are included in the article/**Supplementary Material**. Further inquiries can be directed to the corresponding author.

## Supplementary material

Supplementary Figure 3 The flowchart of our research process.

Supplementary Figure 4 Expression of the risk gene AL031775.1 in different cell subpopulations. **(A, B)** The t-SNE plots show the annotation of these 8 different cell populations, which include various cell types such as CD4Tconv, CD8Tex, endothelial cells, fibroblasts, malignant cells, mono/giant cells, osteoblasts, and plasma cells. **(C, D)** Proportion of different cell types among all cells and proportion of distribution of cell types in different patient samples. **(E, F)** The risk gene AL031775.1 was highly expressed in a variety of immune cells including CD4Tconv, CD8Tex, Mono/Macro, and plasma cells.

Supplementary Figure 5
**(A)** Co-expression network between TEXRGs and T-cell exhaustion-related lncRNAs. **(B)** In our provided cohort of 32 patients, the Kaplan-Meyer survival curves effectively showed that patients in the red high-risk group had a much lower overall survival rate compared to the blue low-risk group. **(C)** Enrichment by TEXRGs to some pathways with significant immunomodulatory functions, KEGG: PD-L1 expression and PD-1 checkpoint pathway in cancer, JAK-STAT signaling pathway; GO: MHC protein complex, regulation of adaptive immune response, regulation of T cell mediated immunity.

Supplementary Figure 6 We added a blank control group to the original experiment to further demonstrate that the empty plasmid itself did not affect the expression of AL031775.1. **(A, B)** The expression of AL031775.1 was significantly reduced in the MG63 and U2OS cell lines. **(C–E)** AL031775.1 expression was significantly increased in the MG63, U2OS and 143b cell lines.
