# Supplementary figures and images for: Identification and functional characterization of T-cell exhaustion-associated lncRNA AL031775.1 in osteosarcoma: a novel therapeutic target
Source: Front Immunol. 2025 Feb 24;16:1517971. doi: 10.3389/fimmu.2025.1517971 (PMC11891247; doi:10.3389/fimmu.2025.1517971)

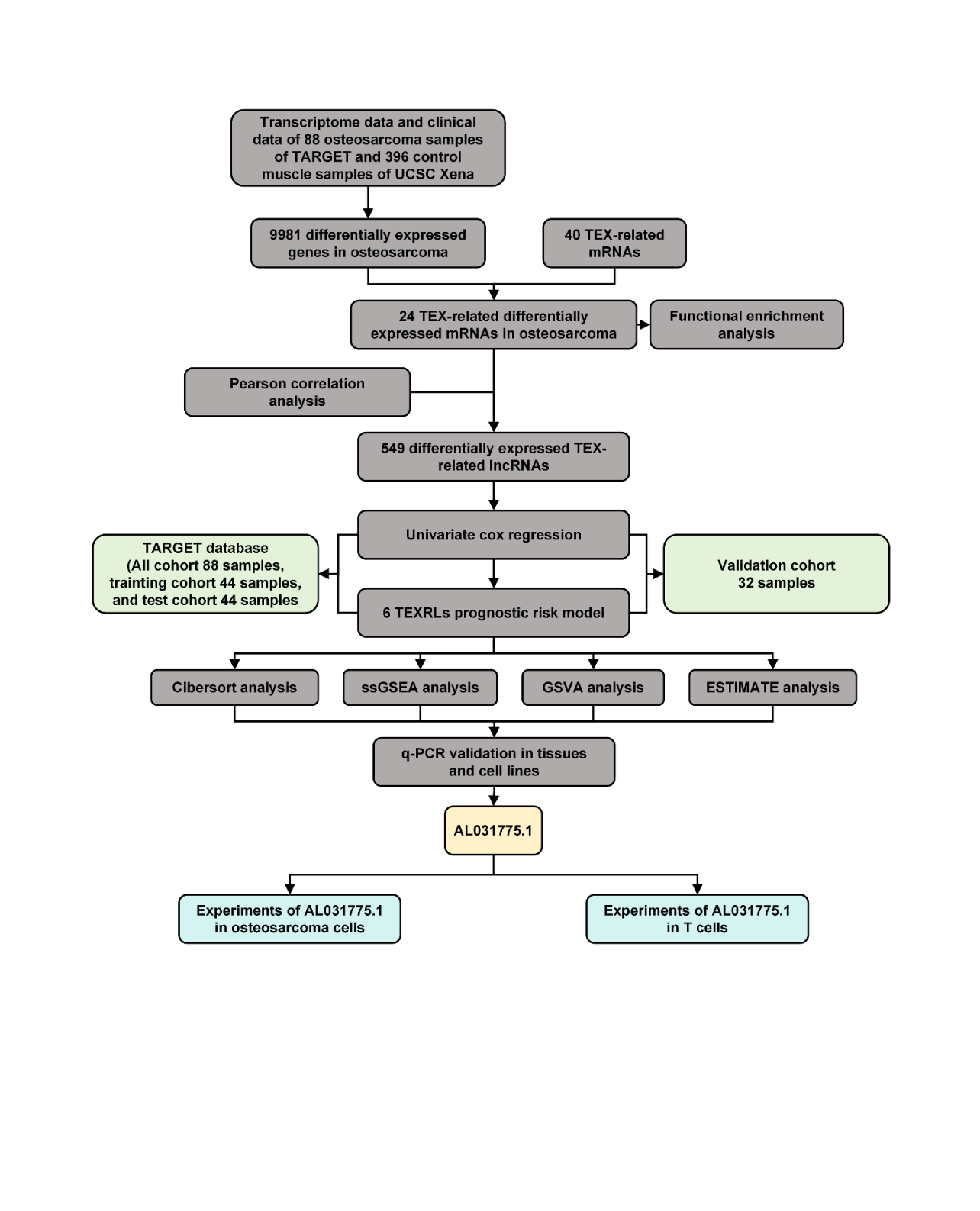

Supplement: Supplementary Figure 3 — The flowchart of our research process. [file Image1.tif]

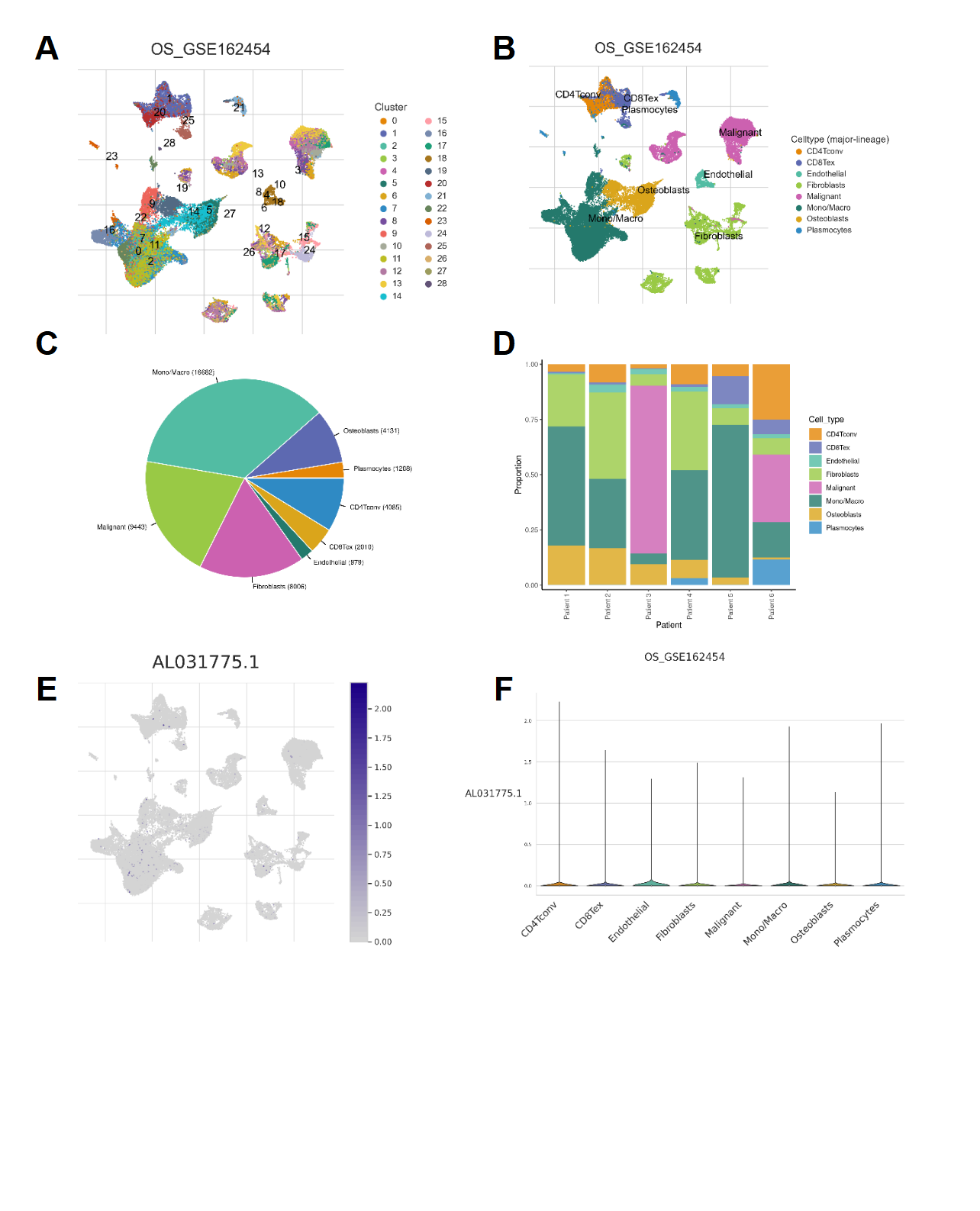

Supplement: Supplementary Figure 4 — Expression of the risk gene AL031775.1 in different cell subpopulations. (A, B) The t-SNE plots show the annotation of these 8 different cell populations, which include various cell types such as CD4Tconv, CD8Tex, endothelial cells, fibroblasts, malignant cells, mono/giant cells, osteoblasts, and plasma cells. (C, D) Proportion of different cell types among all cells and proportion of distribution of cell types in different patient samples. (E, F) The risk gene AL031775.1 was highly expressed in a variety of immune cells including CD4Tconv, CD8Tex, Mono/Macro, and plasma cells. [file Image2.tif]

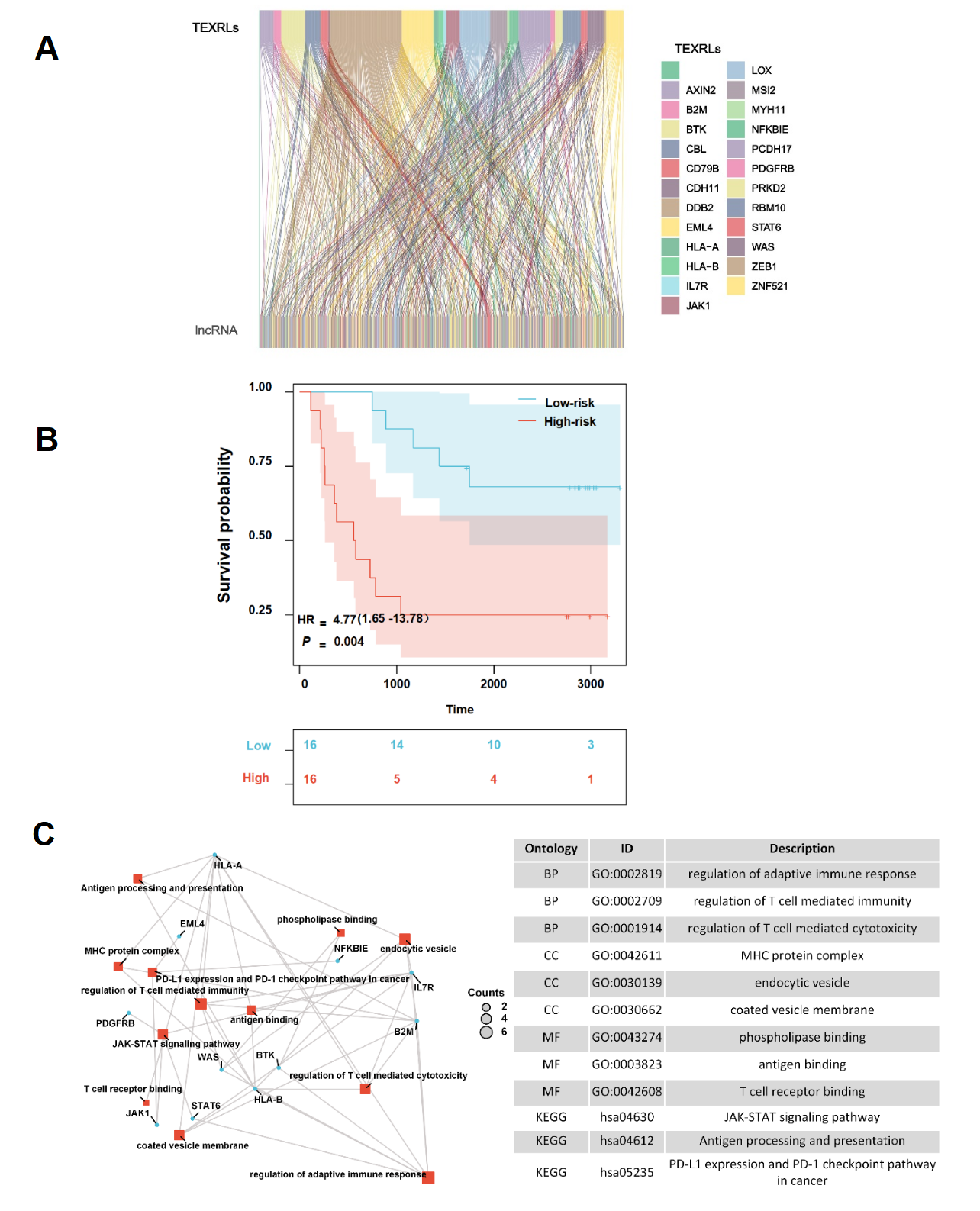

Supplement: Supplementary Figure 5 — (A) Co-expression network between TEXRGs and T-cell exhaustion-related lncRNAs. (B) In our provided cohort of 32 patients, the Kaplan-Meyer survival curves effectively showed that patients in the red high-risk group had a much lower overall survival rate compared to the blue low-risk group. (C) Enrichment by TEXRGs to some pathways with significant immunomodulatory functions, KEGG: PD-L1 expression and PD-1 checkpoint pathway in cancer, JAK-STAT signaling pathway; GO: MHC protein complex, regulation of adaptive immune response, regulation of T cell mediated immunity. [file Image3.tif]

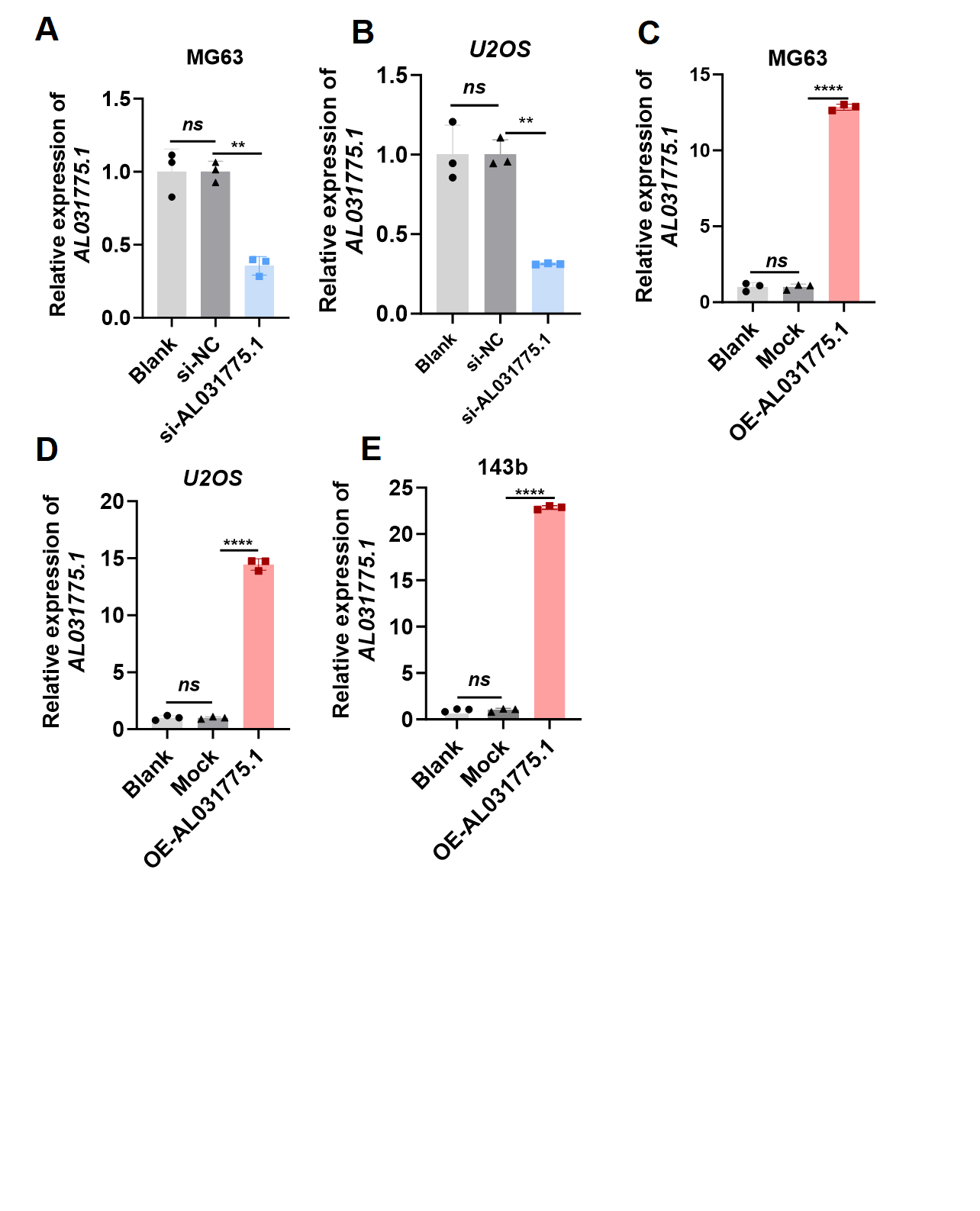

Supplement: Supplementary Figure 6 — We added a blank control group to the original experiment to further demonstrate that the empty plasmid itself did not affect the expression of AL031775.1. (A, B) The expression of AL031775.1 was significantly reduced in the MG63 and U2OS cell lines. (C–E) AL031775.1 expression was significantly increased in the MG63, U2OS and 143b cell lines. [file Image4.tif]
