# Supplementary material for: Identification and functional characterization of T-cell exhaustion-associated lncRNA AL031775.1 in osteosarcoma: a novel therapeutic target
Source: Front Immunol. 2025 Feb 24;16:1517971. doi: 10.3389/fimmu.2025.1517971 (PMC11891247; doi:10.3389/fimmu.2025.1517971)
Supplement: Supplementary file 5 [file Table1.docx]

Supplementary Material 1

# Supplementary Figures and Tables

##
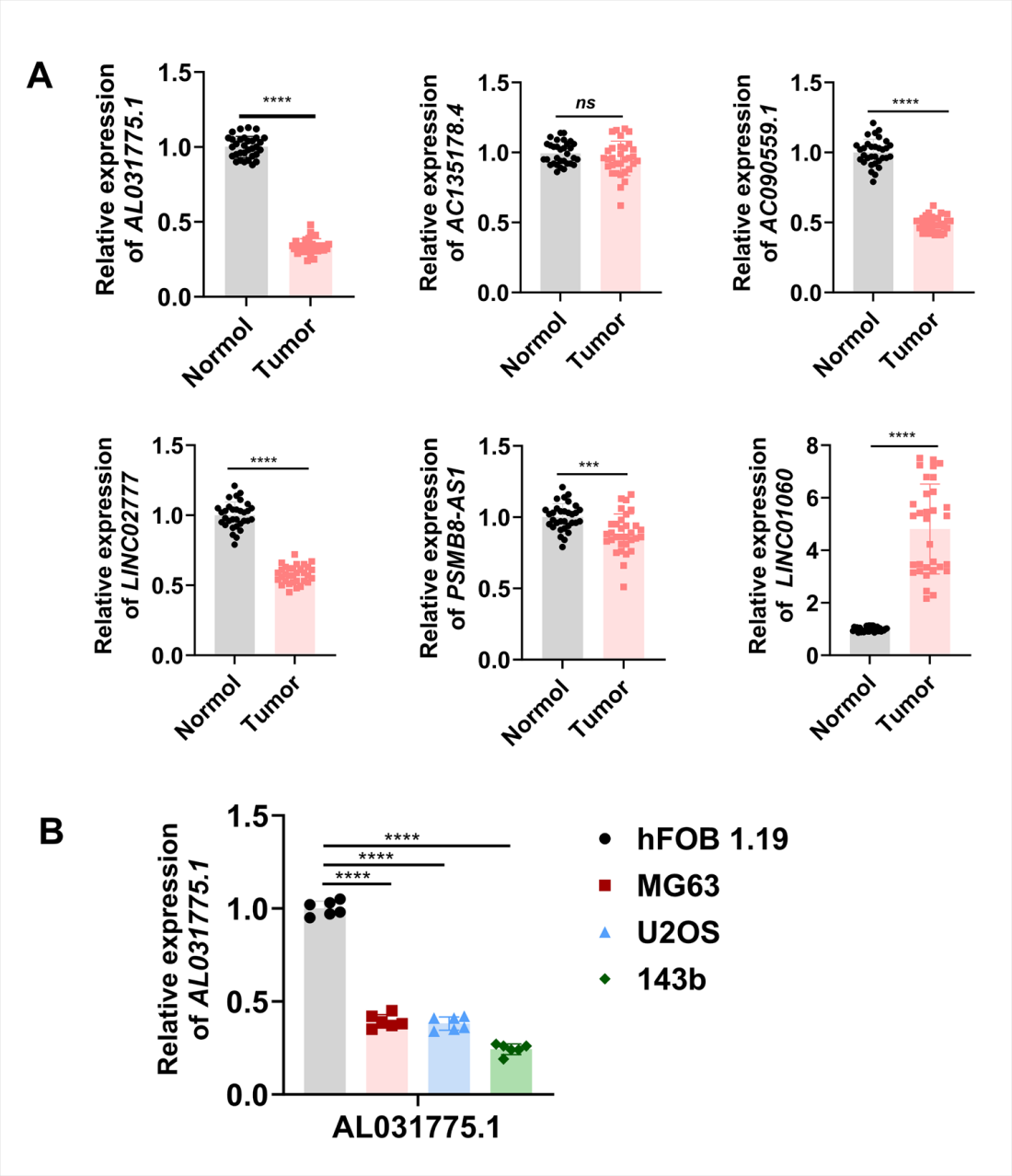
Supplementary Figures

**Supplementary FIGURE 1**

**The expression of AL031775.1 in tumor samples and osteosarcoma cell lines. (A)** AC090559.1, AL031775.1, and LINC02777 were significantly downregulated in the osteosarcoma samples, while LINC01060 exhibited notable upregulation. No significant differential expression was observed in PSMB8-AS1 or AC135178.4. **(B)** Expression of AL031775.1 was significantly downregulated in three osteosarcoma cell lines.


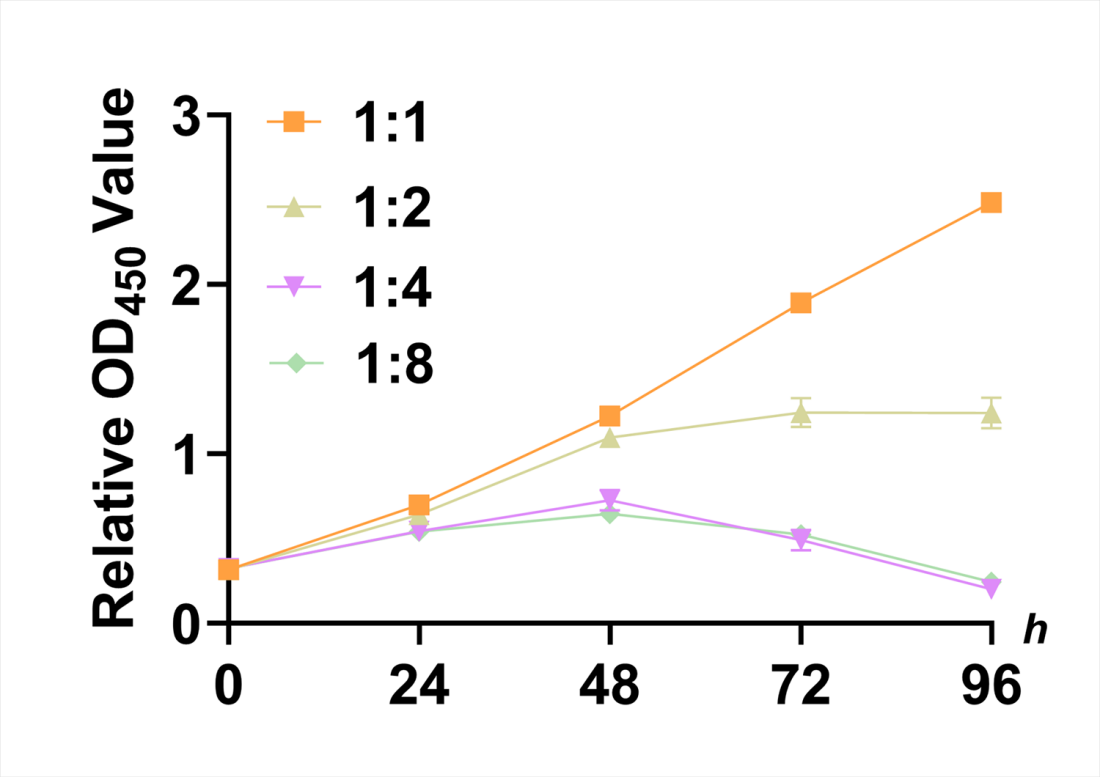


**Supplementary FIGURE 2**

**Analysis of the cytotoxic capacity of T cells.** T cells were co-cultured with 143B osteosarcoma cells at varying ratios (143B cell ratios of 1:1, 1:2, 1:4, and 1:8). Following 48 hours of co-culture, the optical density (OD) values of the residual tumor cells were measured using the CCK-8 assay to assess T cell cytotoxicity. The optimal killing efficiency was observed at a 1:4 ratio.

## Supplementary Tables

**Table S1**

Primers used for RT-qPCR analysis of lncRNAs levels.

| Target ID | Primer sequence 5’-3’ |
| --- | --- |
| h-AL031775.1 | F: CTCCTAATTTTGCAGGTGACATG  R: TGTGAGATGCACACAACTTTCC |
| AC090559.1 | F: CACGCAGAGGAGCACG  R: ATCGTGCTGGAATGTGGCT |
| PSMB8-AS1 | F: GGAAAGACATCGGACCGTCA  R: TCGACAGTTGCTGGGTAGATG |
| LINC01060 | F: CCCGAAAGGAAGAAGCTATACG  R: TGCGACACTTTATCTAATGAGTGTG |
| GAPDH | F: GGAGCGAGATCCCTCCAAAAT  R: GGCTGTTGTCATACTTCTCATGG |

**Table S1**

See Figure1 raw data
