# Supplementary material for: Identification and functional characterization of T-cell exhaustion-associated lncRNA AL031775.1 in osteosarcoma: a novel therapeutic target
Source: Front Immunol. 2025 Feb 24;16:1517971. doi: 10.3389/fimmu.2025.1517971 (PMC11891247; doi:10.3389/fimmu.2025.1517971)
Supplement: Supplementary file 6 [file Table2.docx]

Supplementary Material 2

# Supplementary Data

All original data in the manuscript can be accessed [https://www.jianguoyun.com/p/DccfASQQjdeBDRiPlegFIAA](https://www.jianguoyun.com/p/DccfASQQjdeBDRiPlegFIAA).
